# Four pyrrole derivatives used as building blocks in the synthesis of minor-groove binders

**DOI:** 10.1107/S2056989017001177

**Published:** 2017-01-27

**Authors:** Alan R. Kennedy, Abedawn I. Khalaf, Fraser J. Scott, Colin J. Suckling

**Affiliations:** a Westchem, Department of Pure & Applied Chemistry, University of Strathclyde, 295 Cathedral Street, Glasgow G1 1XL, Scotland; b School of Chemistry, University of Lincoln, Brayford Pool, Lincoln LN6 7TS, England

**Keywords:** crystal structure, nitro­pyrrole, minor-groove binders, hydrogen bonding

## Abstract

The title nitro­pyrrole-based compounds, C_7_H_8_N_2_O_4_, (I) (ethyl 4-nitro-1*H*-pyrrole-2-carboxyl­ate), its derivative C_12_H_14_N_2_O_4_, (II) [ethyl 4-nitro-1-(4-pent­yn­yl)-1*H*-pyrrole-2-carboxyl­ate], C_15_H_26_N_4_O_3_, (III) {*N*-[3-(di­methyamino)prop­yl]-1-isopentyl-4-nitro-1*H*-pyrrole-2-carboxamide}, and C_20_H_27_N_9_O_5_, (IV) {1-(3-azido­prop­yl)-4-(1-methyl-4-nitro-1*H*-pyrrole-2-carboxamido)-*N*-[2-(morpholin-4-yl)eth­yl]-1*H*-pyrrole-2-carboxamide}, are inter­mediates used in the synthesis of modified DNA minor-groove binders. In all four compounds, the nitro groups lie in the plane of the pyrrole ring. In compounds (I) and (II), the ester groups also lie in the plane of the pyrrole ring. In compound (III), both of the other substituents lie out of the plane of the pyrrole ring. In the case of compound (IV), the coplanarity extends to the second pyrrole ring and through both amide groups. In the crystals of all four compounds, layer-like structures are formed, *via* a combination of N—H⋯O and C—H⋯O hydrogen bonds for (I), (III) and (IV), but by only C—H⋯O hydrogen bonds for (II).

## Abstract 2

The title nitro­pyrrole-based compounds are inter­mediates used in the synthesis of modified DNA minor-groove binders. They are ethyl 4-nitro-1*H*-pyrrole-2-carboxyl­ate, its derivative ethyl 4-nitro-1-(4-pentyn­yl)-1*H*-pyrrole-2-carboxyl­ate, *N*-[3-(di­methyl­amino)­prop­yl]-1-isopentyl-4-nitro-1*H*-pyrrole-2-carboxamide and 1-(3-azido­prop­yl)-4-(1-methyl-4-nitro-1*H*-pyrrole-2-carboxamido)-*N*-[2-(morpholin-4-yl)eth­yl]-1*H*-pyrrole-2-carboxamide.

## Chemical context   

Over the past two decades, the field of minor-groove binders (MGBs) has expanded vastly and now these compounds display a wide spectrum of biological activities, such as anti­bacterial, anti­fungal, anti­parasitic and anti­cancer activities. A large number of structural modifications have been carried out on the original, naturally occurring compounds distamycin and netropsin, in order to optimize their biological activities (Lang *et al.*, 2014 ▸). In addition to modifying the biological activities, structural changes have been made to the head group, tail group and the heterocyclic moieties in order to modulate their solubility, selectivity and the degree of binding to the minor groove of DNA (Alniss *et al.*, 2014 ▸). We have recently turned to developing MGB-biotin hybrid mol­ecules to be used as novel biochemical probes in order to determine the mechanism of action of MGBs. Structural information is important in this field, as inter­molecular contacts are important for minor-groove binding and mol­ecular conformation is relevant to structure–activity and model building (Chenoweth & Dervan, 2009 ▸). This paper details the crystal structures of a number of key building blocks that have facilitated this mol­ecular probe development.

## Structural commentary   

**Figure 1:**
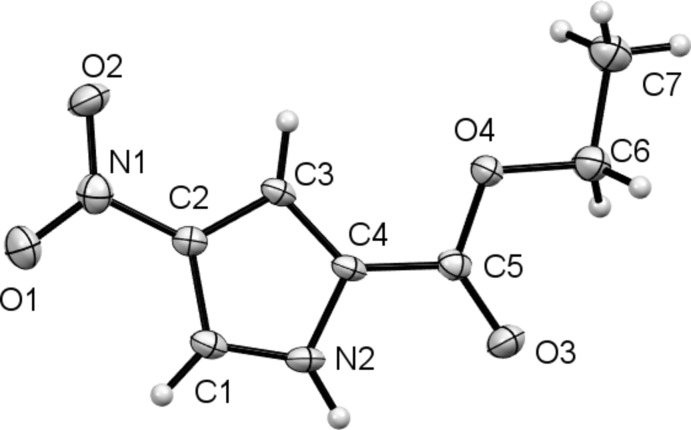
The mol­ecular structure of compound (I), with the atom labelling and 50% probability displacement ellipsoids.

Compound (I), illustrated in Fig. 1 ▸, was produced as an inter­mediate in the synthesis of ethyl 4-nitro-1-(4-pentyn­yl)-1*H*-pyrrole-2-carboxyl­ate (II). Its mol­ecular structure is essentially planar with both the nitro and the ester functionalities coplanar with the pyrrole ring; torsion angles O1—N1—C2—C1 and N2—C4—C5—O3 are −1.5 (4) and 4.4 (4)°, respectively.
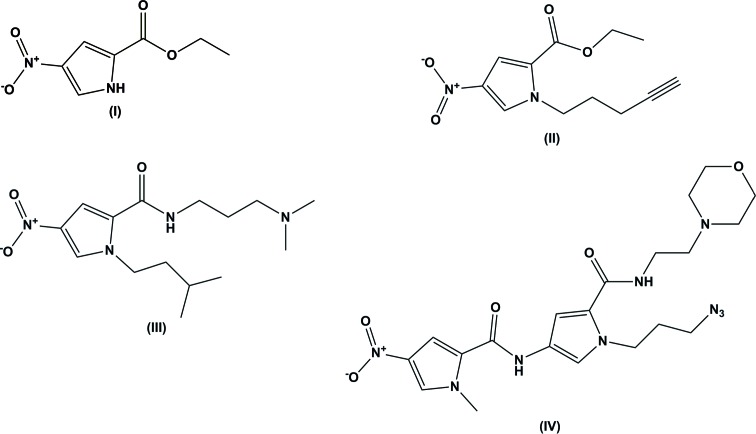


**Figure 2:**
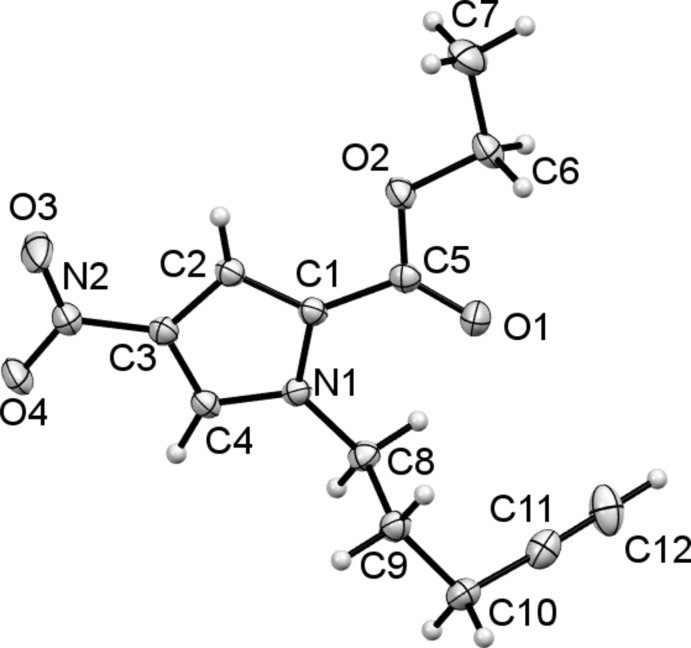
The mol­ecular structure of compound (II), with the atom labelling and 50% probability displacement ellipsoids.

Compound (II), illustrated in Fig. 2 ▸, is an alkyne-functionalized derivative of (I) which allows for late stage diversification, and introduction of biological probe moieties, such as biotin, through application of robust click-chemistry methods. As with (I), the nitro and ester groups are approximately coplanar with the plane of the pyrrole ring. Here torsion angles O4—N2—C3—C2 and N1—C1—C5—O1 are 178.43 (14) and −8.1 (2)°, respectively. However, the overall planarity of the mol­ecule is broken by the pentynyl function, with torsion angle C1—N1—C8—C9 being 86.21 (17)°.

**Figure 3:**
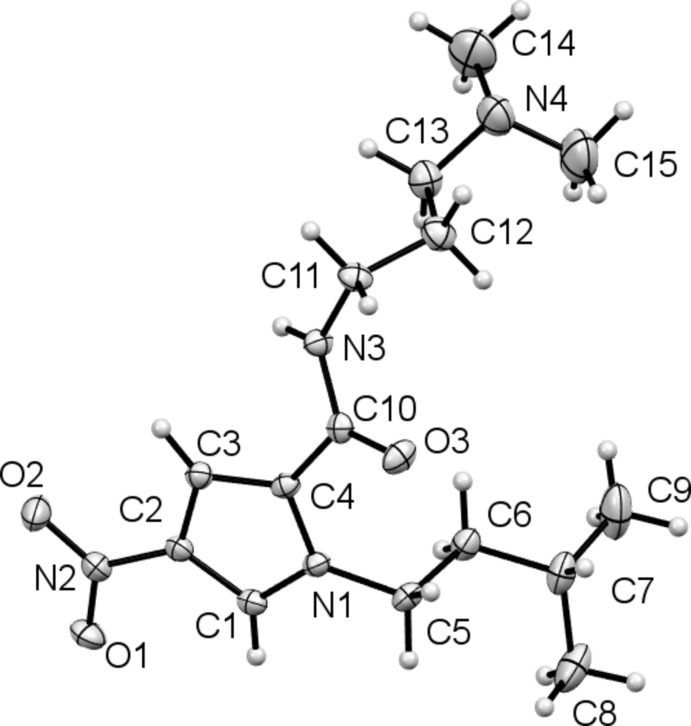
The mol­ecular structure of compound (III), with the atom labelling and 50% probability displacement ellipsoids.

The mol­ecular structure of compound (III) is shown in Fig. 3 ▸. It has the same 4-nitro pyrrole core as compounds (I) and (II) but has an amide substituent rather than an ester, and the pyrrole N atom now bears an iso-pentyl fragment. The introduction of the basic tail group, in this case the di­methyl­amino­propyl moiety, is a crucial feature for biological activity in these MGBs. The nitro group is again coplanar with the pyrrole ring, with torsion angle O2—N2—C2—C1 = 179.34 (15)°, but both the other substituents lie out of the plane of the pyrrole ring.

**Figure 4:**
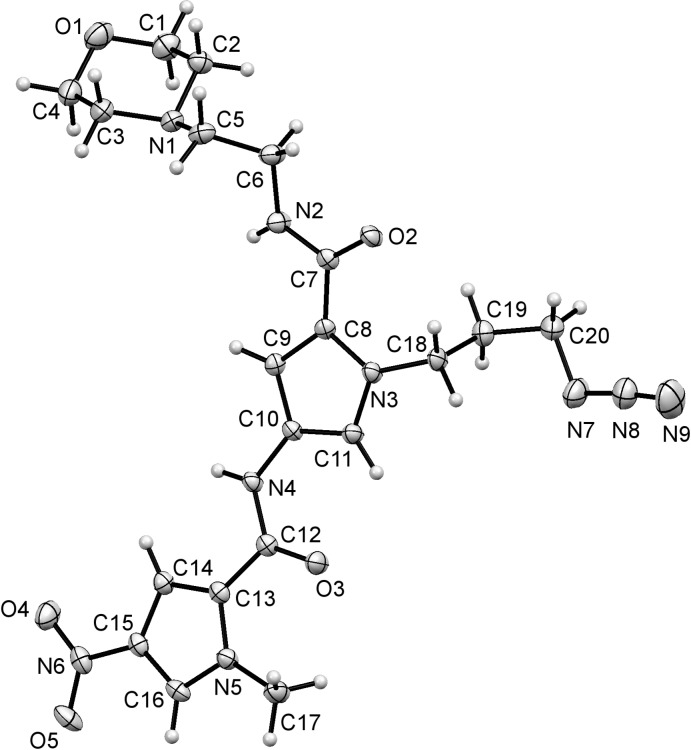
The mol­ecular structure of compound (IV), with the atom labelling and 50% probability displacement ellipsoids.

The final structure reported, compound (IV), is illustrated in Fig. 4 ▸. It is another example of a compound containing a moiety that can be functionalized with click chemistry, this time an azide. Here, there are two pyrrole rings present, one of which is a 4-nitro pyrrole as found in compounds (I), (II) and (III). As with the previous structures, the nitro group is essentially coplanar with the pyrrole ring [torsion angle O4—N6—C15—C14 = −2.8 (3)°] and this coplanarity extends to the second pyrrole ring and through both amide groups [torsion angles O3—C12—C13—N5, C12—N4—C10—C11 and O2—C7—C8—N3 are 3.1 (3), 5.5 (3) and −2.9 (3)°, respectively]. The amide O atoms and the pyrrole N atoms are all mutually *syn* with respect to the mol­ecular axis running through them.

## Supra­molecular features   

**Figure 5:**
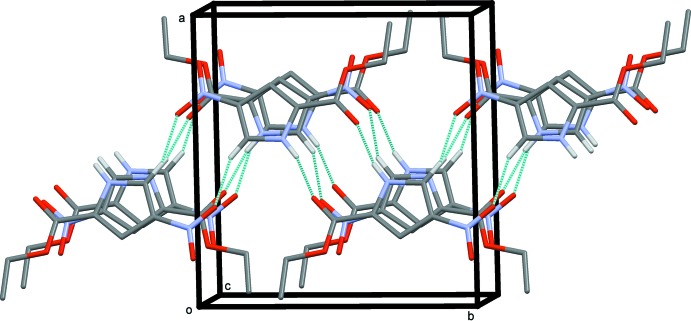
The crystal packing of compound (I), viewed along the *c* axis. The inter­molecular interactions (See Table 1 ▸) are shown as dashed lines. For clarity, only the H atoms involved in these inter­actions have been included.

In the crystal of (I), a primary hydrogen-bonding inter­action is formed, as would be expected, between the N—H donor and the carbonyl acceptor. This gives a centrosymmetric 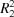
(10) motif. A weaker secondary centrosymmetric 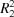
(10) hydrogen-bonding motif is also present; see Fig. 5 ▸ and Table 1 ▸. This is formed by a pyrrole C—H donor and an O atom of the nitro group. Both hydrogen-bonded ring motifs are approximately coplanar with mol­ecular (I) and thus a two-dimensional supra­molecular structure results with layers of mol­ecules parallel to plane (10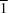). Inter­actions between the layers are both through dipole-to-dipole contacts [nitro-to-carbonyl N⋯C distance = 3.174 (4) Å] and through π–π contacts [closest C-to-C distance, C1⋯C4, is 3.304 (4) Å]. The layered structure of (I) seems to be reflected in its crystal morphology. The samples were stacked thin plates. An approximately single sample was obtained by cutting – but some degree of non-single nature is reflected in the slightly high *R* factors and the higher than expected residual electron density.

**Figure 6:**
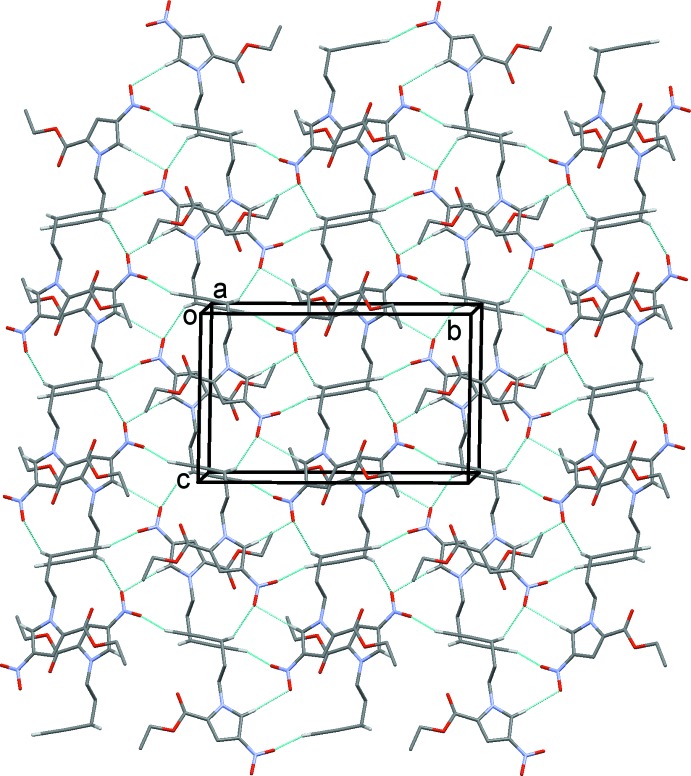
The crystal packing of compound (II), viewed along the *a* axis. The inter­molecular interactions (See Table 2 ▸) are shown as dashed lines. For clarity, only the H atoms involved in these inter­actions have been included.

In the crystal of (II), as no strong hydrogen-bond donor is present, the supra­molecular contacts are limited to non-classical C—H⋯O hydrogen bonds (Table 2 ▸ and Fig. 6 ▸), which combine to give layers parallel to the *bc* plane, and π–π contacts [C5⋯C4^i^ = 3.319 (2) Å; symmetry code: (i) 2 − *x*, −*y*, 1 − *z*] that link the layers. In contrast to (I) there are no dipole–dipole-type contacts involving the nitro group and, perhaps surprisingly, the carbonyl group is not involved in the inter­molecular hydrogen bonding. There is a short intra­molecular contact [O1⋯C8 = 2.925 (2), O1⋯H8*A* = 2.41 Å] which may disfavour inter­molecular bonding here.

**Figure 7:**
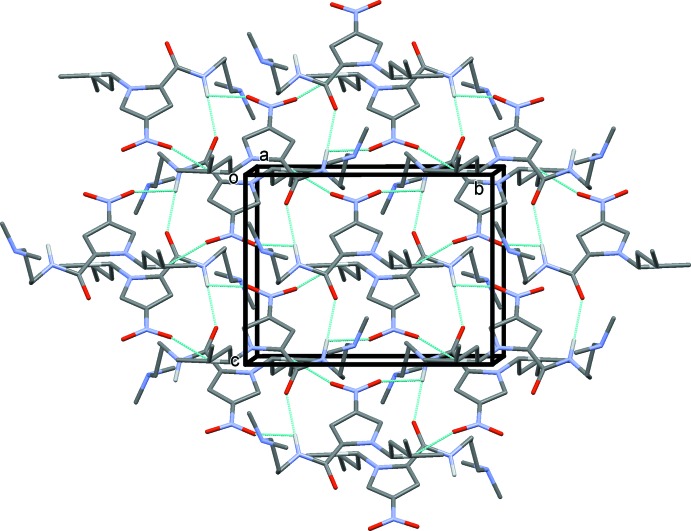
The crystal packing of compound (III), viewed along the *a* axis. The inter­molecular interactions (See Table 3 ▸) are shown as dashed lines. For clarity, only the H atoms involved in these inter­actions have been included.

In the crystal of (III), the amide N—H group can be described as acting as a bifurcated donor giving two hydrogen bonds (Table 3 ▸ and Fig. 7 ▸), forming a short contact with the amide C=O group and a much longer contact to an O atom of a nitro group. These combine to give an 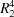
(16) motif, shown in Fig. 7 ▸. The carbonyl group also makes an intra­molecular C—H-to-O contact similar to that found in the structure of (II) [O3⋯C5 = 2.970 (2), O3⋯H5*A* = 2.40 Å; see Table 4 ▸]; however, here, with a strong N—H hydrogen-bond donor available, this is not enough to prevent O3 taking part in other contacts. The structure of (III), composed of hydrogen-bonded layers parallel to the *bc* plane, features no short π–π or dipole–dipole contacts.

**Figure 8:**
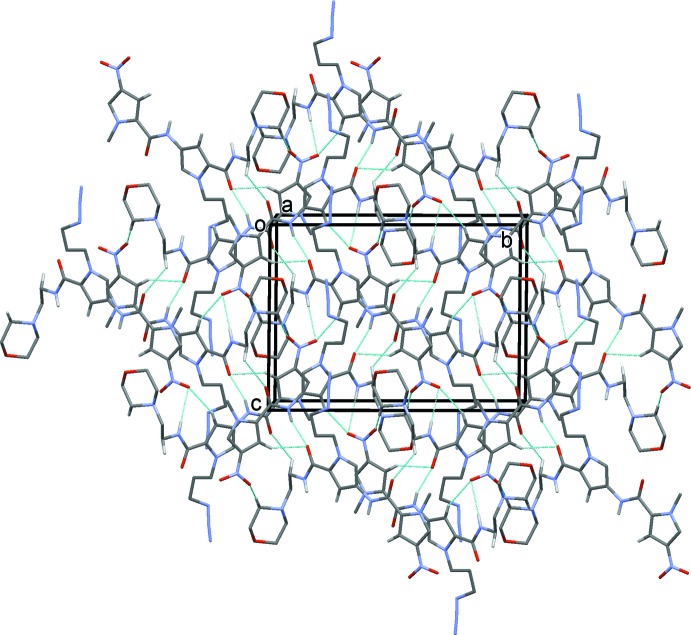
The crystal packing of compound (IV), viewed along the *a* axis. The inter­molecular interactions (See Table 4 ▸) are shown as dashed lines. For clarity, only the H atoms involved in these inter­actions have been included.

In the crystal of (IV), there are two classical N—H⋯O hydrogen bonds (Table 4 ▸ and Fig. 8 ▸) that involve both of the amide N—H groups, but surprisingly only one of the potential amide C=O acceptors. The other acceptor O atom is O5 of the nitro group. These hydrogen bonds combine to give layers parallel to the *bc* plane. As with (II), the reason for the second amide carbonyl group not acting as a classical hydrogen-bond acceptor may lie with a short intra­molecular contact [O3⋯C11 = 2.765 (3) Å, O3⋯H11 = 2.27 Å; see Table 4 ▸]. The remaining shortest inter­molecular contact involves the terminal N atom of the N_3_ group. This forms a short contact with the methyl carbon C17 [N9⋯C17^ii^ 2.968 (3) Å; symmetry code: (ii) = −*x* + 1, −*y*, −*z* + 1) and these contacts form the primary bridges between the layers described above.

## Database survey   

A search of the Cambridge Structural Database (Version 5.37, update May 2016; Groom *et al.*, 2016 ▸) yielded zero hits for 4-nitro­pyrrole-2-carboxyl­ates and only 12 hits for 4-nitro­pyrrole-2-carboxamides. One of the latter, *viz.* dimeth­yl{3-[1-methyl-4-(1-methyl-4-nitro­pyrrole-2-carboxamido)­pyrrole-2-carboxamido]­prop­yl}ammonium chloride methanol solvate (RACBAZ; Lu *et al.*, 2003 ▸), has a (4-nitro­pyrrole-2-carboxamido)­pyrrole-2-carboxamide unit present, as in compound (IV). Here, the conformation of this unit is slightly more planar than that for compound (IV). For example, the two pyrrole rings are inclined to one another by 3.7 (2)° compared to 9.3 (1)° in compound (IV).

## Synthesis and crystallization   

**Ethyl 4-nitro-1** ***H*** **-pyrrole-2-carboxyl­ate (I)**. 4-Nitro-1*H*-pyrrole-2-carb­oxy­lic acid was dissolved in thionyl chloride (10 mL) and heated under reflux for 2 h. Excess thionyl chloride was removed under reduced pressure and the acid chloride so formed was dissolved in di­chloro­methane (25 mL, dry) to which ethanol (10 mL) and TEA (2 mL) were added. The stirring was continued at room temperature overnight. Solvent and excess reagents were removed under reduced pressure and the residue was partitioned between brine (50 mL) and ethyl acetate (100 mL). After the extraction, the water layer was extracted again with ethyl acetate (2 × 100 mL). The combined organic extracts were dried (Na_2_SO_4_), filtered and the solvent removed under reduced pressure. The crude product obtained was applied to a silica gel column and eluted with 1/2 ethyl acetate/*n*-hexane. The required product was obtained as a brown solid (1.070 g, 93%), m.p. 445–447 K [reference m.p. 447–448 K, Lee *et al.*, 1988 ▸]. IR: 750, 775, 808, 841, 961, 1017, 1086, 1119, 1148, 1204, 1263, 1316, 1364, 1383, 1420, 14670, 1503, 1566, 1684, 3264 cm^−1^. ^1^H NMR (DMSO-*d*_6_): 9.81(1H, *br*), 7.77(1H, *dd*, *J* = 3.5 Hz & *J* = 1.6 Hz), 7.41(1H, *dd*, *J* = 2.6 Hz & *J* = 1.8 Hz), 4.41(2H, *qt*, *J* = 7.1 Hz), 1.4(3H, *q*, *J* = 7.1 Hz). HRESIMS: found 185.0555; calculated 185.0557.

**Ethyl 4-nitro-1-(4-pentyn­yl)-1** ***H*** **-pyrrole-2-carboxyl­ate (II)**. Ethyl 4-nitro-1*H*-pyrrole-2-carboxyl­ate (0.230 g, 1.25 mmol) was dissolved in acetone (25 mL) to which sodium carbonate (0.395 g, 3.73 mmol), tetra­butyl­ammonium iodide (0.462 g, 1.25 mmol), and propyl bromide solution 80 weight % in toluene (1.50 mL) were added. The reaction mixture was heated under reflux for 6 h after which time it was left stirring at room temperature overnight. Water and ethyl acetate were added to the reaction mixture. After extraction, the organic layers were collected, dried (Na_2_SO_4_), filtered and the solvent removed under reduced pressure. The crude product was applied to a silica gel column and eluted with (1/4 ethyl acetate/*n*-hexane, *R*_F_ = 0.35). The required product was obtained as a white solid (0.270 g, 83%), m.p. 335–337 K [It was obtained as a colourless oil by Satam *et al.*, 2014 ▸]. IR: 754, 808, 864, 1018, 1084, 1107, 1165, 1188, 1250, 1285, 1312, 1364, 1383, 1422, 1497, 1533, 1717 cm^−1^. ^1^H NMR (CDCl_3_): 7.70 (1H, *d*, *J* = 2.0 Hz), 7.46 (1H, *d*, *J* = 2.0 Hz), 4.53 (2H, *t*, *J* = 6.8 Hz), 4.35 (2H, *q*, *J* = 7.2 Hz), 2.24 (2H, *dt*, *J* = 6.7 Hz & *J* = 2.7 Hz), 2.09 (1H, *t*, *J* = 2.7 Hz), 2.07 (2H, *qt*, *J* = 6.7 Hz), 1.40 (3H, *t*, *J* = 7.1 Hz). HRESIMS: found 251.1010; calculated 251.1026.

***N*** **-[3-(Di­methyl­amino)­prop­yl]-1-isopentyl-4-nitro-1** ***H*** **-pyrrole-2-carboxamide (III)**. Following Khalaf *et al.*, 2004 ▸, 4-nitro-*N*-isopropyl-pyrrole-2-carb­oxy­lic acid (0.315g, 1.39 mmol) was dissolved in thionyl chloride (5 mL) and heated at reflux for 4 h. The excess thionyl chloride was removed under reduced pressure at 323 K to give the acid chloride as a white solid that was used without further purification. 3-(Di­methyl­amino)­propyl­amine (0.25 mL, 2.47 mmol) was dissolved in THF (20 mL, dry) to which *N*-methyl­morpholine (0.25 mL) was added at room temperature with stirring. The acid chloride was dissolved in THF (5 mL, dry) and added dropwise to the amine solution at room temperature with stirring. The reaction mixture was then left stirring at room temperature overnight. Following this, the solvent was removed under reduced pressure at 323 K and then the crude product was extracted with aqueous potassium carbonate solution (25 mL, 10% *w*/*v*) and di­chloro­methane (2 × 50 mL). The organic layer was collected, dried (Na_2_SO_4_), and filtered, and the solvent was removed under reduced pressure. The crude product was purified by chromatography over silica gel using 100:100:1 methanol/ethyl acetate/tri­ethyl­amine to give the required product as a pale-yellow solid (410 mg, 95%), m.p. 345–346 K. IR (KBr): 1656, 1637, 1565, 1534, 1498, 1417, 1333 cm^−1^. ^1^H NMR (CDCl_3_): 0.95 (6H, *d*, *J* = 6.5 Hz), 1.57–1.76 (5H, *m*), 2.32 (6H, *s*), 2.51 (2H, *t*, *J* = 10.3 Hz), 3.47–3.51 (2H, *quintet*, *J* = 4.8 Hz), 4.40–4.44 (2H, *q*, *J* = 7.5 Hz), 6.92 (1H, *d*, *J* = 1.9 Hz), 7.56 (1H, *d*, *J* = 1.9 Hz), 8.61 (1H, *s*, *br*, CONH). HRESIMS: found 310.20031; calculated 310.20049.

**1-(3-Azido­prop­yl)-4-(1-methyl-4-nitro-1** ***H*** **-pyrrole-2-carboxamido)-** ***N*** **-[2-(morpholin-4-yl)eth­yl]-1** ***H*** **-pyrrole-2-carboxamide (IV)**. 1-(3-chloro­prop­yl)-4-(1-methyl-4-nitro-1*H*-pyrrole-2-carboxamido)-*N*-(2-morpholino­eth­yl)-1*H*-pyrrole-2-carboxamide (100 mg, 0.214 mmol) was dissolved in DMF (5 mL, anhydrous) to which was added sodium azide (41.7 mg, 0.642 mmol). This solution was heated at 333 K overnight with stirring and then the DMF was removed *in vacuo*. The resulting residue was dissolved in ethyl acetate (10 mL), washed with water (3 x 10 mL) and the organic layer was reduced in volume by rotary evaporation to approximately 1 mL and the product was obtained as a crystalline solid after several hours (81 mg, 80%). IR: 3357, 3294, 3140, 2954, 2857, 2805, 2097, 1617, 1496, 1303, 1115 cm^−1^. ^1^H NMR (DMSO): 10.26 (1H, *s*), 8.18 (1H, *d*, *J* = 1.6 Hz), 8.00 (1H, *t*, *J* = 5.6 Hz), 7.58 (1H, *d*, *J* = 1.6 Hz), 7.27 (1H, *d*, *J* = 1.6 Hz), 6.85 (1H, *d*, *J* = 1.6 Hz), 4.34 (2H, *t*, *J* = 6.4 Hz), 3.96 (3H, *s*), 3.58 (4H, *t*, *J* = 4.4 Hz), 3.25–3.30 (4H, *m*), 2.40–2.45 (6H, *m*), 1.93 (2H, *pentet*, *J* = 6.8Hz). HRESIMS: found 474.2202; calculated 474.2208.

## Refinement   

Crystal data, data collection and structure refinement details are summarized in Table 5 ▸. The H atoms bound to N were located in difference Fourier maps and freely refined for (I) and (IV). In compound (III), the N—H distance was restrained to be 0.93 (1) Å. For all structures, C-bound H atoms were placed in the expected geometrical positions and treated as riding: C—H = 0.95–0.99 Å with *U*_iso_(H) = 1.5*U*_eq_(C-meth­yl) and 1.2*U*_eq_(C) for other H atoms.

## Supplementary Material

Crystal structure: contains datablock(s) I, II, III, IV, global. DOI: 10.1107/S2056989017001177/su5346sup1.cif

Structure factors: contains datablock(s) I. DOI: 10.1107/S2056989017001177/su5346Isup2.hkl

Structure factors: contains datablock(s) II. DOI: 10.1107/S2056989017001177/su5346IIsup3.hkl

Structure factors: contains datablock(s) III. DOI: 10.1107/S2056989017001177/su5346IIIsup4.hkl

Structure factors: contains datablock(s) IV. DOI: 10.1107/S2056989017001177/su5346IVsup5.hkl

Supporting information file. DOI: 10.1107/S2056989017001177/su5346Isup6.cml

Supporting information file. DOI: 10.1107/S2056989017001177/su5346IIsup7.cml

Supporting information file. DOI: 10.1107/S2056989017001177/su5346IIIsup8.cml

Supporting information file. DOI: 10.1107/S2056989017001177/su5346IVsup9.cml

CCDC references: 1529248, 1529247, 1529246, 1529245

Additional supporting information:  crystallographic information; 3D view; checkCIF report

## Figures and Tables

**Table 1:** Hydrogen-bond geometry (Å, °) for (I)

*D*—H⋯*A*	*D*—H	H⋯*A*	*D*⋯*A*	*D*—H⋯*A*
N2—H1*N*⋯O3^i^	0.90 (4)	2.00 (5)	2.872 (3)	163 (4)
C1—H1⋯O1^ii^	0.95	2.34	3.203 (4)	151

Symmetry codes: (i) 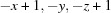; (ii) 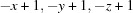.

**Table 2:** Hydrogen-bond geometry (Å, °) for (II)

*D*—H⋯*A*	*D*—H	H⋯*A*	*D*⋯*A*	*D*—H⋯*A*
C4—H4⋯O3^i^	0.95	2.53	3.323 (2)	141
C10—H10*B*⋯O3^ii^	0.99	2.51	3.337 (2)	141
C12—H12⋯O4^iii^	0.95	2.40	3.262 (2)	151

Symmetry codes: (i) 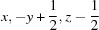; (ii) 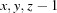; (iii) 
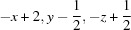
.

**Table 3:** Hydrogen-bond geometry (Å, °) for (III)

*D*—H⋯*A*	*D*—H	H⋯*A*	*D*⋯*A*	*D*—H⋯*A*
N3—H1*N*⋯O3^i^	0.91 (1)	2.01 (1)	2.895 (2)	165 (2)
C5—H5*A*⋯O2^ii^	0.99	2.54	3.460 (2)	154

Symmetry codes: (i) 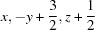; (ii) 
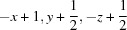
.

**Table 4:** Hydrogen-bond geometry (Å, °) for (IV)

*D*—H⋯*A*	*D*—H	H⋯*A*	*D*⋯*A*	*D*—H⋯*A*
N2—H1*N*⋯O5^i^	0.83 (2)	2.36 (2)	3.176 (2)	171 (2)
N4—H2*N*⋯O2^ii^	0.88 (2)	2.02 (2)	2.864 (2)	162 (2)
C2—H2*A*⋯O4^iii^	0.99	2.53	3.498 (3)	165
C6—H6*B*⋯O3^iv^	0.99	2.58	3.354 (3)	135
C9—H9⋯O5^i^	0.95	2.43	3.322 (2)	156
C14—H14⋯O2^ii^	0.95	2.46	3.317 (3)	149

Symmetry codes: (i) 
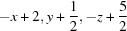
; (ii) 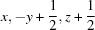; (iii) 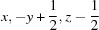; (iv) 
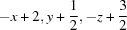
.

**Table 5:** Experimental details

	(I)	(II)	(III)	(IV)
Crystal data				
Chemical formula	C_7_H_8_N_2_O_4_	C_12_H_14_N_2_O_4_	C_15_H_26_N_4_O_3_	C_20_H_27_N_9_O_5_
*M*_r_	184.15	250.25	310.40	473.51
Crystal system, space group	Monoclinic, *P*2_1_/*c*	Monoclinic, *P*2_1_/*c*	Monoclinic, *P*2_1_/*c*	Monoclinic, *P*2_1_/*c*
Temperature (K)	123	123	123	123
*a*, *b*, *c* (Å)	11.0318 (13), 10.4108 (13), 7.1659 (8)	7.8839 (4), 16.1443 (7), 10.2058 (5)	17.5744 (7), 11.3718 (6), 8.7299 (4)	11.2809 (4), 16.4528 (6), 12.5130 (5)
β (°)	96.734 (10)	104.472 (5)	92.076 (4)	106.542 (4)
*V* (Å^3^)	817.32 (17)	1257.78 (10)	1743.55 (14)	2226.32 (14)
*Z*	4	4	4	4
Radiation type	Mo *K*α	Mo *K*α	Mo *K*α	Mo *K*α
μ (mm^−1^)	0.13	0.10	0.08	0.11
Crystal size (mm)	0.35 × 0.25 × 0.02	0.38 × 0.14 × 0.06	0.40 × 0.30 × 0.04	0.30 × 0.28 × 0.03

Data collection				
Diffractometer	Oxford Diffraction Xcalibur E	Oxford Diffraction Xcalibur E	Oxford Diffraction Xcalibur E	Oxford Diffraction Xcalibur E
Absorption correction	Multi-scan (*CrysAlis PRO*; Oxford Diffraction, 2010 ▸)	Multi-scan (*CrysAlis PRO*; Oxford Diffraction, 2010 ▸)	Multi-scan (*CrysAlis PRO*; Oxford Diffraction, 2010 ▸)	Multi-scan (*CrysAlis PRO*; Oxford Diffraction, 2010 ▸)
*T*_min_, *T*_max_	0.679, 1.000	0.918, 1.000	0.995, 1.000	0.828, 1.000
No. of measured, independent and observed [*I* > 2σ(*I*)] reflections	4995, 1604, 1240	6098, 2745, 2133	8252, 3971, 2873	14949, 4852, 3295
*R*_int_	0.038	0.025	0.030	0.038
(sin θ/λ)_max_ (Å^−1^)	0.617	0.639	0.650	0.639

Refinement				
*R*[*F* ^2^ > 2σ(*F* ^2^)], *wR*(*F* ^2^), *S*	0.073, 0.210, 1.17	0.041, 0.100, 1.03	0.053, 0.145, 1.03	0.049, 0.128, 1.03
No. of reflections	1604	2745	3971	4852
No. of parameters	123	164	206	316
No. of restraints	0	0	1	0
H-atom treatment	H atoms treated by a mixture of independent and constrained refinement	H-atom parameters constrained	H atoms treated by a mixture of independent and constrained refinement	H atoms treated by a mixture of independent and constrained refinement
Δρ_max_, Δρ_min_ (e Å^−3^)	0.73, −0.30	0.22, −0.24	0.33, −0.27	0.29, −0.32

Computer programs: *CrysAlis PRO* (Agilent, 2014 ▸), *SIR92* (Altomare *et al.*, 1994 ▸), *SHELXL97* (Sheldrick, 2008 ▸) and *Mercury* (Macrae *et al.*, 2008 ▸).

## supplementary crystallographic information

### (I) Ethyl 4-nitro-1H-pyrrole-2-carboxylate Crystal data

**Table:**

C_7_H_8_N_2_O_4_	*F*(000) = 384
*M**_r_* = 184.15	*D*_x_ = 1.497 Mg m^−^^3^
Monoclinic, *P*2_1_/*c*	Mo *K*α radiation, λ = 0.71073 Å
*a* = 11.0318 (13) Å	Cell parameters from 1975 reflections
*b* = 10.4108 (13) Å	θ = 3.2–28.3°
*c* = 7.1659 (8) Å	µ = 0.13 mm^−^^1^
β = 96.734 (10)°	*T* = 123 K
*V* = 817.32 (17) Å^3^	Plate, colourless
*Z* = 4	0.35 × 0.25 × 0.02 mm

### (I) Ethyl 4-nitro-1H-pyrrole-2-carboxylate Data collection

**Table:**

Oxford Diffraction Xcalibur E diffractometer	1604 independent reflections
Radiation source: fine-focus sealed tube	1240 reflections with *I* > 2σ(*I*)
Graphite monochromator	*R*_int_ = 0.038
ω scans	θ_max_ = 26.0°, θ_min_ = 3.5°
Absorption correction: multi-scan (CrysAlis PRO; Oxford Diffraction, 2010)	*h* = −13→13
*T*_min_ = 0.679, *T*_max_ = 1.000	*k* = −11→12
4995 measured reflections	*l* = −8→8

### (I) Ethyl 4-nitro-1H-pyrrole-2-carboxylate Refinement

**Table:**

Refinement on *F*^2^	Primary atom site location: structure-invariant direct methods
Least-squares matrix: full	Secondary atom site location: difference Fourier map
*R*[*F*^2^ > 2σ(*F*^2^)] = 0.073	Hydrogen site location: inferred from neighbouring sites
*wR*(*F*^2^) = 0.210	H atoms treated by a mixture of independent and constrained refinement
*S* = 1.17	*w* = 1/[σ^2^(*F*_o_^2^) + (0.1052*P*)^2^ + 0.5276*P*] where *P* = (*F*_o_^2^ + 2*F*_c_^2^)/3
1604 reflections	(Δ/σ)_max_ = 0.001
123 parameters	Δρ_max_ = 0.73 e Å^−^^3^
0 restraints	Δρ_min_ = −0.30 e Å^−^^3^

### (I) Ethyl 4-nitro-1H-pyrrole-2-carboxylate Special details

**Table:**

Geometry. All s.u.'s (except the s.u. in the dihedral angle between two l.s. planes) are estimated using the full covariance matrix. The cell s.u.'s are taken into account individually in the estimation of s.u.'s in distances, angles and torsion angles; correlations between s.u.'s in cell parameters are only used when they are defined by crystal symmetry. An approximate (isotropic) treatment of cell s.u.'s is used for estimating s.u.'s involving l.s. planes.
Refinement. Refinement of F^2^ against ALL reflections. The weighted R-factor wR and goodness of fit S are based on F^2^, conventional R-factors R are based on F, with F set to zero for negative F^2^. The threshold expression of F^2^ > 2σ(F^2^) is used only for calculating R-factors(gt) etc. and is not relevant to the choice of reflections for refinement. R-factors based on F^2^ are statistically about twice as large as those based on F, and R- factors based on ALL data will be even larger.

### (I) Ethyl 4-nitro-1H-pyrrole-2-carboxylate Fractional atomic coordinates and isotropic or equivalent isotropic displacement parameters (Å^2^)

**Table:**

	*x*	*y*	*z*	*U*_iso_*/*U*_eq_	
O1	0.6535 (2)	0.5757 (2)	0.6758 (3)	0.0334 (7)	
O2	0.8216 (2)	0.5067 (2)	0.8325 (3)	0.0321 (6)	
O3	0.6242 (2)	−0.0697 (2)	0.6309 (3)	0.0284 (6)	
O4	0.79942 (18)	−0.0218 (2)	0.8128 (3)	0.0225 (6)	
N1	0.7216 (2)	0.4878 (3)	0.7390 (3)	0.0254 (7)	
N2	0.5700 (2)	0.1948 (3)	0.6026 (3)	0.0211 (6)	
C1	0.5726 (3)	0.3226 (3)	0.6068 (4)	0.0203 (7)	
H1	0.5102	0.3788	0.5530	0.024*	
C2	0.6831 (3)	0.3584 (3)	0.7041 (4)	0.0189 (7)	
C3	0.7498 (3)	0.2472 (3)	0.7609 (4)	0.0181 (7)	
H3	0.8287	0.2428	0.8300	0.022*	
C4	0.6769 (2)	0.1465 (3)	0.6954 (4)	0.0179 (7)	
C5	0.6964 (3)	0.0084 (3)	0.7088 (4)	0.0192 (7)	
C6	0.8251 (3)	−0.1596 (3)	0.8261 (5)	0.0290 (8)	
H6A	0.8214	−0.1978	0.6991	0.035*	
H6B	0.7645	−0.2034	0.8954	0.035*	
C7	0.9511 (3)	−0.1739 (4)	0.9291 (5)	0.0325 (9)	
H7A	1.0100	−0.1298	0.8592	0.049*	
H7B	0.9720	−0.2653	0.9406	0.049*	
H7C	0.9533	−0.1360	1.0546	0.049*	
H1N	0.513 (4)	0.143 (4)	0.546 (6)	0.055 (13)*	

### (I) Ethyl 4-nitro-1H-pyrrole-2-carboxylate Atomic displacement parameters (Å^2^)

**Table:**

	*U*^11^	*U*^22^	*U*^33^	*U*^12^	*U*^13^	*U*^23^
O1	0.0340 (14)	0.0233 (14)	0.0415 (14)	0.0062 (10)	−0.0014 (10)	0.0016 (10)
O2	0.0239 (12)	0.0294 (14)	0.0408 (13)	−0.0063 (10)	−0.0050 (10)	−0.0058 (10)
O3	0.0227 (12)	0.0247 (13)	0.0359 (13)	−0.0045 (10)	−0.0039 (9)	−0.0034 (10)
O4	0.0213 (11)	0.0189 (12)	0.0253 (11)	0.0015 (9)	−0.0053 (8)	0.0002 (9)
N1	0.0263 (14)	0.0236 (15)	0.0270 (14)	0.0008 (12)	0.0057 (11)	0.0002 (11)
N2	0.0139 (12)	0.0293 (16)	0.0189 (12)	−0.0021 (11)	−0.0031 (9)	−0.0010 (11)
C1	0.0167 (14)	0.0271 (18)	0.0164 (14)	0.0020 (13)	−0.0010 (11)	0.0031 (12)
C2	0.0165 (14)	0.0216 (17)	0.0182 (14)	0.0004 (12)	−0.0002 (11)	−0.0002 (12)
C3	0.0134 (14)	0.0238 (17)	0.0166 (13)	0.0028 (11)	−0.0007 (11)	0.0024 (11)
C4	0.0136 (13)	0.0249 (17)	0.0144 (13)	0.0017 (12)	−0.0019 (10)	0.0029 (12)
C5	0.0181 (14)	0.0230 (17)	0.0165 (13)	−0.0004 (13)	0.0015 (11)	0.0020 (12)
C6	0.0251 (17)	0.0252 (18)	0.0348 (18)	0.0019 (14)	−0.0047 (13)	−0.0023 (14)
C7	0.0238 (17)	0.033 (2)	0.0397 (19)	0.0033 (15)	−0.0013 (14)	0.0006 (15)

### (I) Ethyl 4-nitro-1H-pyrrole-2-carboxylate Geometric parameters (Å, º)

**Table:**

O1—N1	1.236 (3)	C2—C3	1.406 (4)
O2—N1	1.237 (3)	C3—C4	1.371 (4)
O3—C5	1.225 (4)	C3—H3	0.9500
O4—C5	1.322 (3)	C4—C5	1.455 (4)
O4—C6	1.464 (4)	C6—C7	1.502 (4)
N1—C2	1.426 (4)	C6—H6A	0.9900
N2—C1	1.331 (5)	C6—H6B	0.9900
N2—C4	1.380 (4)	C7—H7A	0.9800
N2—H1N	0.90 (4)	C7—H7B	0.9800
C1—C2	1.383 (4)	C7—H7C	0.9800
C1—H1	0.9500		
			
C5—O4—C6	114.6 (2)	C3—C4—C5	131.1 (3)
O1—N1—O2	123.1 (3)	N2—C4—C5	120.3 (3)
O1—N1—C2	118.7 (3)	O3—C5—O4	124.7 (3)
O2—N1—C2	118.2 (3)	O3—C5—C4	122.8 (3)
C1—N2—C4	109.8 (2)	O4—C5—C4	112.5 (2)
C1—N2—H1N	129 (3)	O4—C6—C7	106.9 (3)
C4—N2—H1N	121 (3)	O4—C6—H6A	110.3
N2—C1—C2	107.2 (3)	C7—C6—H6A	110.3
N2—C1—H1	126.4	O4—C6—H6B	110.3
C2—C1—H1	126.4	C7—C6—H6B	110.3
C1—C2—C3	109.0 (3)	H6A—C6—H6B	108.6
C1—C2—N1	124.7 (3)	C6—C7—H7A	109.5
C3—C2—N1	126.3 (3)	C6—C7—H7B	109.5
C4—C3—C2	105.3 (3)	H7A—C7—H7B	109.5
C4—C3—H3	127.3	C6—C7—H7C	109.5
C2—C3—H3	127.3	H7A—C7—H7C	109.5
C3—C4—N2	108.7 (3)	H7B—C7—H7C	109.5
			
C4—N2—C1—C2	0.0 (3)	C2—C3—C4—C5	179.4 (3)
N2—C1—C2—C3	0.0 (3)	C1—N2—C4—C3	0.0 (3)
N2—C1—C2—N1	179.8 (3)	C1—N2—C4—C5	−179.5 (2)
O1—N1—C2—C1	−1.5 (4)	C6—O4—C5—O3	1.7 (4)
O2—N1—C2—C1	178.1 (3)	C6—O4—C5—C4	−178.3 (2)
O1—N1—C2—C3	178.3 (3)	C3—C4—C5—O3	−175.0 (3)
O2—N1—C2—C3	−2.1 (4)	N2—C4—C5—O3	4.4 (4)
C1—C2—C3—C4	0.1 (3)	C3—C4—C5—O4	4.9 (4)
N1—C2—C3—C4	−179.8 (3)	N2—C4—C5—O4	−175.7 (2)
C2—C3—C4—N2	−0.1 (3)	C5—O4—C6—C7	173.4 (3)

### (I) Ethyl 4-nitro-1H-pyrrole-2-carboxylate Hydrogen-bond geometry (Å, º)

**Table:**

*D*—H···*A*	*D*—H	H···*A*	*D*···*A*	*D*—H···*A*
N2—H1*N*···O3^i^	0.90 (4)	2.00 (5)	2.872 (3)	163 (4)
C1—H1···O1^ii^	0.95	2.34	3.203 (4)	151

Symmetry codes: (i) −*x*+1, −*y*, −*z*+1; (ii) −*x*+1, −*y*+1, −*z*+1.

### (II) Ethyl 4-nitro-1-(4-pentynyl)-1H-pyrrole-2-carboxylate Crystal data

**Table:**

C_12_H_14_N_2_O_4_	*F*(000) = 528
*M**_r_* = 250.25	*D*_x_ = 1.322 Mg m^−^^3^
Monoclinic, *P*2_1_/*c*	Mo *K*α radiation, λ = 0.71073 Å
Hall symbol: -P 2ybc	Cell parameters from 2521 reflections
*a* = 7.8839 (4) Å	θ = 3.2–29.3°
*b* = 16.1443 (7) Å	µ = 0.10 mm^−^^1^
*c* = 10.2058 (5) Å	*T* = 123 K
β = 104.472 (5)°	Rod, colourless
*V* = 1257.78 (10) Å^3^	0.38 × 0.14 × 0.06 mm
*Z* = 4	

### (II) Ethyl 4-nitro-1-(4-pentynyl)-1H-pyrrole-2-carboxylate Data collection

**Table:**

Oxford Diffraction Xcalibur E diffractometer	2745 independent reflections
Radiation source: fine-focus sealed tube	2133 reflections with *I* > 2σ(*I*)
Graphite monochromator	*R*_int_ = 0.025
ω scans	θ_max_ = 27.0°, θ_min_ = 3.2°
Absorption correction: multi-scan (CrysAlis PRO; Oxford Diffraction, 2010)	*h* = −10→9
*T*_min_ = 0.918, *T*_max_ = 1.000	*k* = −20→17
6098 measured reflections	*l* = −13→12

### (II) Ethyl 4-nitro-1-(4-pentynyl)-1H-pyrrole-2-carboxylate Refinement

**Table:**

Refinement on *F*^2^	Primary atom site location: structure-invariant direct methods
Least-squares matrix: full	Secondary atom site location: difference Fourier map
*R*[*F*^2^ > 2σ(*F*^2^)] = 0.041	Hydrogen site location: inferred from neighbouring sites
*wR*(*F*^2^) = 0.100	H-atom parameters constrained
*S* = 1.03	*w* = 1/[σ^2^(*F*_o_^2^) + (0.0359*P*)^2^ + 0.3332*P*] where *P* = (*F*_o_^2^ + 2*F*_c_^2^)/3
2745 reflections	(Δ/σ)_max_ < 0.001
164 parameters	Δρ_max_ = 0.22 e Å^−^^3^
0 restraints	Δρ_min_ = −0.24 e Å^−^^3^

### (II) Ethyl 4-nitro-1-(4-pentynyl)-1H-pyrrole-2-carboxylate Special details

**Table:**

Geometry. All s.u.'s (except the s.u. in the dihedral angle between two l.s. planes) are estimated using the full covariance matrix. The cell s.u.'s are taken into account individually in the estimation of s.u.'s in distances, angles and torsion angles; correlations between s.u.'s in cell parameters are only used when they are defined by crystal symmetry. An approximate (isotropic) treatment of cell s.u.'s is used for estimating s.u.'s involving l.s. planes.
Refinement. Refinement of F^2^ against ALL reflections. The weighted R-factor wR and goodness of fit S are based on F^2^, conventional R-factors R are based on F, with F set to zero for negative F^2^. The threshold expression of F^2^ > 2σ(F^2^) is used only for calculating R-factors(gt) etc. and is not relevant to the choice of reflections for refinement. R-factors based on F^2^ are statistically about twice as large as those based on F, and R- factors based on ALL data will be even larger.

### (II) Ethyl 4-nitro-1-(4-pentynyl)-1H-pyrrole-2-carboxylate Fractional atomic coordinates and isotropic or equivalent isotropic displacement parameters (Å^2^)

**Table:**

	*x*	*y*	*z*	*U*_iso_*/*U*_eq_	
O1	0.67236 (15)	−0.08852 (7)	0.29527 (11)	0.0320 (3)	
O2	0.66323 (13)	−0.10194 (6)	0.51291 (11)	0.0242 (3)	
O3	0.8949 (2)	0.18072 (8)	0.77978 (13)	0.0489 (4)	
O4	1.00887 (15)	0.25558 (7)	0.64857 (12)	0.0325 (3)	
N1	0.84828 (15)	0.07104 (8)	0.37170 (12)	0.0192 (3)	
N2	0.93141 (17)	0.19300 (8)	0.67172 (14)	0.0261 (3)	
C1	0.77664 (17)	0.01969 (9)	0.45352 (15)	0.0190 (3)	
C2	0.79676 (18)	0.05720 (9)	0.57710 (15)	0.0204 (3)	
H2	0.7608	0.0365	0.6530	0.024*	
C3	0.88142 (18)	0.13247 (9)	0.56798 (15)	0.0203 (3)	
C4	0.91140 (18)	0.13959 (9)	0.44135 (15)	0.0201 (3)	
H4	0.9667	0.1848	0.4091	0.024*	
C5	0.69928 (18)	−0.06124 (9)	0.40866 (15)	0.0210 (3)	
C6	0.5915 (2)	−0.18454 (9)	0.48267 (17)	0.0276 (4)	
H6A	0.6748	−0.2201	0.4501	0.033*	
H6B	0.4800	−0.1822	0.4117	0.033*	
C7	0.5610 (2)	−0.21857 (11)	0.61188 (19)	0.0391 (5)	
H7A	0.6723	−0.2205	0.6811	0.059*	
H7B	0.5123	−0.2746	0.5959	0.059*	
H7C	0.4785	−0.1828	0.6430	0.059*	
C8	0.86099 (19)	0.05609 (10)	0.23232 (15)	0.0222 (3)	
H8A	0.8812	−0.0037	0.2205	0.027*	
H8B	0.9626	0.0869	0.2164	0.027*	
C9	0.69648 (19)	0.08289 (10)	0.12910 (15)	0.0237 (3)	
H9A	0.6743	0.1423	0.1423	0.028*	
H9B	0.5953	0.0509	0.1431	0.028*	
C10	0.7135 (2)	0.06919 (10)	−0.01578 (16)	0.0285 (4)	
H10A	0.6022	0.0852	−0.0801	0.034*	
H10B	0.8069	0.1057	−0.0323	0.034*	
C11	0.75444 (19)	−0.01676 (11)	−0.04200 (16)	0.0285 (4)	
C12	0.7889 (2)	−0.08626 (12)	−0.0585 (2)	0.0377 (4)	
H12	0.8167	−0.1424	−0.0718	0.045*	

### (II) Ethyl 4-nitro-1-(4-pentynyl)-1H-pyrrole-2-carboxylate Atomic displacement parameters (Å^2^)

**Table:**

	*U*^11^	*U*^22^	*U*^33^	*U*^12^	*U*^13^	*U*^23^
O1	0.0447 (7)	0.0269 (6)	0.0227 (6)	−0.0082 (5)	0.0050 (5)	−0.0042 (5)
O2	0.0289 (5)	0.0186 (5)	0.0261 (6)	−0.0061 (4)	0.0089 (5)	−0.0016 (5)
O3	0.0915 (11)	0.0352 (8)	0.0284 (7)	−0.0224 (7)	0.0306 (7)	−0.0113 (6)
O4	0.0432 (7)	0.0192 (6)	0.0359 (7)	−0.0106 (5)	0.0112 (5)	−0.0032 (5)
N1	0.0202 (6)	0.0199 (6)	0.0171 (7)	0.0005 (5)	0.0038 (5)	0.0017 (5)
N2	0.0341 (7)	0.0188 (7)	0.0256 (8)	−0.0030 (6)	0.0078 (6)	−0.0017 (6)
C1	0.0194 (7)	0.0173 (7)	0.0197 (8)	0.0006 (6)	0.0037 (6)	0.0021 (6)
C2	0.0224 (7)	0.0180 (7)	0.0212 (8)	0.0011 (6)	0.0064 (6)	0.0017 (6)
C3	0.0215 (7)	0.0177 (7)	0.0208 (8)	0.0011 (6)	0.0033 (6)	0.0007 (6)
C4	0.0195 (7)	0.0162 (7)	0.0237 (8)	−0.0013 (6)	0.0040 (6)	0.0017 (6)
C5	0.0200 (7)	0.0201 (8)	0.0223 (8)	0.0023 (6)	0.0040 (6)	0.0006 (6)
C6	0.0293 (8)	0.0173 (8)	0.0356 (10)	−0.0048 (6)	0.0072 (7)	−0.0019 (7)
C7	0.0462 (10)	0.0295 (10)	0.0397 (11)	−0.0126 (8)	0.0073 (8)	0.0070 (8)
C8	0.0244 (7)	0.0247 (8)	0.0184 (8)	0.0011 (6)	0.0069 (6)	−0.0003 (6)
C9	0.0266 (7)	0.0234 (8)	0.0200 (8)	0.0036 (6)	0.0036 (6)	0.0004 (6)
C10	0.0357 (9)	0.0288 (9)	0.0192 (8)	0.0015 (7)	0.0035 (7)	0.0010 (7)
C11	0.0243 (8)	0.0374 (10)	0.0238 (9)	−0.0022 (7)	0.0058 (6)	−0.0050 (7)
C12	0.0326 (9)	0.0337 (10)	0.0506 (12)	−0.0030 (8)	0.0173 (8)	−0.0146 (9)

### (II) Ethyl 4-nitro-1-(4-pentynyl)-1H-pyrrole-2-carboxylate Geometric parameters (Å, º)

**Table:**

O1—C5	1.2064 (18)	C6—H6A	0.9900
O2—C5	1.3399 (18)	C6—H6B	0.9900
O2—C6	1.4512 (18)	C7—H7A	0.9800
O3—N2	1.2235 (17)	C7—H7B	0.9800
O4—N2	1.2336 (16)	C7—H7C	0.9800
N1—C4	1.3420 (19)	C8—C9	1.515 (2)
N1—C1	1.3926 (18)	C8—H8A	0.9900
N1—C8	1.4707 (18)	C8—H8B	0.9900
N2—C3	1.4221 (19)	C9—C10	1.534 (2)
C1—C2	1.372 (2)	C9—H9A	0.9900
C1—C5	1.466 (2)	C9—H9B	0.9900
C2—C3	1.401 (2)	C10—C11	1.464 (2)
C2—H2	0.9500	C10—H10A	0.9900
C3—C4	1.376 (2)	C10—H10B	0.9900
C4—H4	0.9500	C11—C12	1.177 (2)
C6—C7	1.502 (2)	C12—H12	0.9500
			
C5—O2—C6	115.49 (12)	H6A—C6—H6B	108.6
C4—N1—C1	109.00 (12)	C6—C7—H7A	109.5
C4—N1—C8	122.81 (12)	C6—C7—H7B	109.5
C1—N1—C8	128.18 (12)	H7A—C7—H7B	109.5
O3—N2—O4	122.99 (13)	C6—C7—H7C	109.5
O3—N2—C3	118.42 (13)	H7A—C7—H7C	109.5
O4—N2—C3	118.59 (13)	H7B—C7—H7C	109.5
C2—C1—N1	108.50 (13)	N1—C8—C9	111.85 (11)
C2—C1—C5	128.60 (13)	N1—C8—H8A	109.2
N1—C1—C5	122.87 (13)	C9—C8—H8A	109.2
C1—C2—C3	105.63 (13)	N1—C8—H8B	109.2
C1—C2—H2	127.2	C9—C8—H8B	109.2
C3—C2—H2	127.2	H8A—C8—H8B	107.9
C4—C3—C2	109.36 (13)	C8—C9—C10	111.30 (12)
C4—C3—N2	124.15 (13)	C8—C9—H9A	109.4
C2—C3—N2	126.49 (14)	C10—C9—H9A	109.4
N1—C4—C3	107.50 (12)	C8—C9—H9B	109.4
N1—C4—H4	126.2	C10—C9—H9B	109.4
C3—C4—H4	126.2	H9A—C9—H9B	108.0
O1—C5—O2	124.15 (14)	C11—C10—C9	112.91 (13)
O1—C5—C1	125.78 (14)	C11—C10—H10A	109.0
O2—C5—C1	110.08 (13)	C9—C10—H10A	109.0
O2—C6—C7	106.78 (13)	C11—C10—H10B	109.0
O2—C6—H6A	110.4	C9—C10—H10B	109.0
C7—C6—H6A	110.4	H10A—C10—H10B	107.8
O2—C6—H6B	110.4	C12—C11—C10	177.77 (19)
C7—C6—H6B	110.4	C11—C12—H12	180.0
			
C4—N1—C1—C2	−0.32 (16)	C2—C3—C4—N1	−0.11 (16)
C8—N1—C1—C2	178.42 (13)	N2—C3—C4—N1	179.47 (13)
C4—N1—C1—C5	−178.80 (13)	C6—O2—C5—O1	1.6 (2)
C8—N1—C1—C5	−0.1 (2)	C6—O2—C5—C1	−177.89 (11)
N1—C1—C2—C3	0.25 (15)	C2—C1—C5—O1	173.80 (15)
C5—C1—C2—C3	178.61 (14)	N1—C1—C5—O1	−8.1 (2)
C1—C2—C3—C4	−0.09 (16)	C2—C1—C5—O2	−6.8 (2)
C1—C2—C3—N2	−179.65 (14)	N1—C1—C5—O2	171.39 (12)
O3—N2—C3—C4	178.63 (15)	C5—O2—C6—C7	−179.97 (13)
O4—N2—C3—C4	−1.1 (2)	C4—N1—C8—C9	−95.21 (16)
O3—N2—C3—C2	−1.9 (2)	C1—N1—C8—C9	86.21 (17)
O4—N2—C3—C2	178.43 (14)	N1—C8—C9—C10	178.43 (12)
C1—N1—C4—C3	0.26 (16)	C8—C9—C10—C11	56.43 (18)
C8—N1—C4—C3	−178.56 (12)	C9—C10—C11—C12	−16 (4)

### (II) Ethyl 4-nitro-1-(4-pentynyl)-1H-pyrrole-2-carboxylate Hydrogen-bond geometry (Å, º)

**Table:**

*D*—H···*A*	*D*—H	H···*A*	*D*···*A*	*D*—H···*A*
C4—H4···O3^i^	0.95	2.53	3.323 (2)	141
C10—H10*B*···O3^ii^	0.99	2.51	3.337 (2)	141
C12—H12···O4^iii^	0.95	2.40	3.262 (2)	151

Symmetry codes: (i) *x*, −*y*+1/2, *z*−1/2; (ii) *x*, *y*, *z*−1; (iii) −*x*+2, *y*−1/2, −*z*+1/2.

### (III) N-[3-(Dimethylamino)propyl]-1-isopentyl-4-nitro-1H-pyrrole-2-carboxamide Crystal data

**Table:**

C_15_H_26_N_4_O_3_	*F*(000) = 672
*M**_r_* = 310.40	*D*_x_ = 1.182 Mg m^−^^3^
Monoclinic, *P*2_1_/*c*	Mo *K*α radiation, λ = 0.71073 Å
Hall symbol: -P 2ybc	Cell parameters from 2708 reflections
*a* = 17.5744 (7) Å	θ = 3.2–28.8°
*b* = 11.3718 (6) Å	µ = 0.08 mm^−^^1^
*c* = 8.7299 (4) Å	*T* = 123 K
β = 92.076 (4)°	Plate, colourless
*V* = 1743.55 (14) Å^3^	0.40 × 0.30 × 0.04 mm
*Z* = 4	

### (III) N-[3-(Dimethylamino)propyl]-1-isopentyl-4-nitro-1H-pyrrole-2-carboxamide Data collection

**Table:**

Oxford Diffraction Xcalibur E diffractometer	3971 independent reflections
Radiation source: fine-focus sealed tube	2873 reflections with *I* > 2σ(*I*)
Graphite monochromator	*R*_int_ = 0.030
ω scans	θ_max_ = 27.5°, θ_min_ = 3.2°
Absorption correction: multi-scan (CrysAlis PRO; Oxford Diffraction, 2010)	*h* = −22→22
*T*_min_ = 0.995, *T*_max_ = 1.000	*k* = −13→14
8252 measured reflections	*l* = −11→10

### (III) N-[3-(Dimethylamino)propyl]-1-isopentyl-4-nitro-1H-pyrrole-2-carboxamide Refinement

**Table:**

Refinement on *F*^2^	Primary atom site location: structure-invariant direct methods
Least-squares matrix: full	Secondary atom site location: difference Fourier map
*R*[*F*^2^ > 2σ(*F*^2^)] = 0.053	Hydrogen site location: inferred from neighbouring sites
*wR*(*F*^2^) = 0.145	H atoms treated by a mixture of independent and constrained refinement
*S* = 1.03	*w* = 1/[σ^2^(*F*_o_^2^) + (0.0606*P*)^2^ + 0.6893*P*] where *P* = (*F*_o_^2^ + 2*F*_c_^2^)/3
3971 reflections	(Δ/σ)_max_ < 0.001
206 parameters	Δρ_max_ = 0.33 e Å^−^^3^
1 restraint	Δρ_min_ = −0.27 e Å^−^^3^

### (III) N-[3-(Dimethylamino)propyl]-1-isopentyl-4-nitro-1H-pyrrole-2-carboxamide Special details

**Table:**

Geometry. All s.u.'s (except the s.u. in the dihedral angle between two l.s. planes) are estimated using the full covariance matrix. The cell s.u.'s are taken into account individually in the estimation of s.u.'s in distances, angles and torsion angles; correlations between s.u.'s in cell parameters are only used when they are defined by crystal symmetry. An approximate (isotropic) treatment of cell s.u.'s is used for estimating s.u.'s involving l.s. planes.
Refinement. Refinement of F^2^ against ALL reflections. The weighted R-factor wR and goodness of fit S are based on F^2^, conventional R-factors R are based on F, with F set to zero for negative F^2^. The threshold expression of F^2^ > 2σ(F^2^) is used only for calculating R-factors(gt) etc. and is not relevant to the choice of reflections for refinement. R-factors based on F^2^ are statistically about twice as large as those based on F, and R- factors based on ALL data will be even larger.

### (III) N-[3-(Dimethylamino)propyl]-1-isopentyl-4-nitro-1H-pyrrole-2-carboxamide Fractional atomic coordinates and isotropic or equivalent isotropic displacement parameters (Å^2^)

**Table:**

	*x*	*y*	*z*	*U*_iso_*/*U*_eq_	
O1	0.43676 (7)	1.01567 (12)	0.38580 (15)	0.0292 (3)	
O2	0.43689 (8)	0.82473 (12)	0.37406 (17)	0.0343 (4)	
O3	0.67675 (7)	0.84955 (11)	−0.16325 (14)	0.0257 (3)	
N1	0.60351 (8)	0.99423 (12)	0.07012 (16)	0.0183 (3)	
N2	0.46097 (8)	0.92217 (13)	0.33535 (17)	0.0226 (3)	
N3	0.67714 (8)	0.70166 (13)	0.01195 (17)	0.0200 (3)	
H1N	0.6676 (11)	0.6851 (17)	0.1111 (12)	0.024*	
N4	0.91585 (10)	0.56415 (18)	0.0944 (2)	0.0422 (5)	
C1	0.55171 (9)	1.02695 (15)	0.1723 (2)	0.0195 (4)	
H1	0.5396	1.1052	0.2008	0.023*	
C2	0.51972 (9)	0.92543 (15)	0.22736 (19)	0.0187 (4)	
C3	0.55292 (9)	0.82769 (15)	0.15765 (19)	0.0191 (4)	
H3	0.5412	0.7472	0.1738	0.023*	
C4	0.60561 (9)	0.87244 (15)	0.06166 (19)	0.0174 (4)	
C5	0.65546 (10)	1.07684 (15)	−0.0027 (2)	0.0213 (4)	
H5A	0.6340	1.1573	0.0008	0.026*	
H5B	0.6606	1.0549	−0.1116	0.026*	
C6	0.73347 (10)	1.07512 (17)	0.0790 (2)	0.0244 (4)	
H6A	0.7536	0.9939	0.0775	0.029*	
H6B	0.7276	1.0976	0.1875	0.029*	
C7	0.79112 (11)	1.15716 (18)	0.0085 (2)	0.0308 (5)	
H7	0.7960	1.1345	−0.1014	0.037*	
C8	0.76633 (14)	1.2851 (2)	0.0139 (3)	0.0456 (6)	
H8A	0.7200	1.2957	−0.0504	0.068*	
H8B	0.8069	1.3352	−0.0242	0.068*	
H8C	0.7562	1.3068	0.1198	0.068*	
C9	0.86909 (13)	1.1411 (3)	0.0907 (3)	0.0517 (7)	
H9A	0.9071	1.1890	0.0398	0.078*	
H9B	0.8839	1.0581	0.0869	0.078*	
H9C	0.8661	1.1659	0.1978	0.078*	
C10	0.65675 (9)	0.80817 (15)	−0.04016 (19)	0.0185 (4)	
C11	0.71803 (10)	0.61821 (16)	−0.0811 (2)	0.0233 (4)	
H11A	0.7028	0.6309	−0.1901	0.028*	
H11B	0.7023	0.5376	−0.0534	0.028*	
C12	0.80398 (11)	0.62648 (19)	−0.0639 (2)	0.0303 (5)	
H12A	0.8268	0.5728	−0.1389	0.036*	
H12B	0.8198	0.7076	−0.0891	0.036*	
C13	0.83521 (11)	0.59597 (19)	0.0947 (2)	0.0318 (5)	
H13A	0.8286	0.6642	0.1634	0.038*	
H13B	0.8059	0.5294	0.1355	0.038*	
C14	0.94033 (15)	0.5120 (3)	0.2399 (3)	0.0619 (8)	
H14A	0.9326	0.5684	0.3228	0.093*	
H14B	0.9944	0.4916	0.2373	0.093*	
H14C	0.9105	0.4408	0.2578	0.093*	
C15	0.96397 (14)	0.6629 (3)	0.0604 (4)	0.0631 (8)	
H15A	0.9505	0.6927	−0.0424	0.095*	
H15B	1.0174	0.6378	0.0645	0.095*	
H15C	0.9567	0.7253	0.1359	0.095*	

### (III) N-[3-(Dimethylamino)propyl]-1-isopentyl-4-nitro-1H-pyrrole-2-carboxamide Atomic displacement parameters (Å^2^)

**Table:**

	*U*^11^	*U*^22^	*U*^33^	*U*^12^	*U*^13^	*U*^23^
O1	0.0269 (7)	0.0263 (7)	0.0346 (8)	0.0097 (6)	0.0057 (6)	−0.0033 (6)
O2	0.0338 (7)	0.0252 (8)	0.0452 (9)	−0.0027 (6)	0.0168 (6)	0.0024 (6)
O3	0.0375 (7)	0.0232 (7)	0.0167 (6)	−0.0023 (6)	0.0043 (5)	0.0010 (5)
N1	0.0191 (7)	0.0147 (7)	0.0207 (7)	−0.0002 (6)	−0.0014 (5)	0.0014 (6)
N2	0.0192 (7)	0.0228 (8)	0.0260 (8)	0.0025 (6)	0.0024 (6)	0.0007 (6)
N3	0.0244 (7)	0.0187 (7)	0.0173 (7)	0.0031 (6)	0.0043 (6)	0.0003 (6)
N4	0.0286 (9)	0.0492 (12)	0.0484 (12)	−0.0016 (8)	−0.0035 (8)	0.0092 (9)
C1	0.0184 (8)	0.0180 (9)	0.0219 (9)	0.0033 (7)	−0.0015 (6)	−0.0014 (7)
C2	0.0158 (7)	0.0198 (9)	0.0205 (8)	0.0023 (7)	−0.0003 (6)	0.0008 (7)
C3	0.0183 (8)	0.0174 (9)	0.0213 (9)	0.0002 (7)	−0.0013 (6)	0.0004 (7)
C4	0.0191 (8)	0.0158 (8)	0.0171 (8)	−0.0001 (7)	−0.0022 (6)	0.0004 (6)
C5	0.0265 (9)	0.0162 (8)	0.0211 (9)	−0.0021 (7)	0.0011 (7)	0.0033 (7)
C6	0.0256 (9)	0.0240 (10)	0.0235 (9)	−0.0056 (8)	−0.0015 (7)	0.0044 (8)
C7	0.0317 (10)	0.0356 (11)	0.0253 (10)	−0.0127 (9)	0.0031 (8)	0.0041 (9)
C8	0.0592 (15)	0.0333 (12)	0.0448 (14)	−0.0203 (11)	0.0108 (11)	0.0026 (10)
C9	0.0332 (12)	0.0749 (19)	0.0469 (14)	−0.0221 (12)	−0.0010 (10)	0.0124 (13)
C10	0.0171 (8)	0.0205 (9)	0.0177 (8)	−0.0029 (7)	−0.0020 (6)	−0.0020 (7)
C11	0.0260 (9)	0.0221 (9)	0.0219 (9)	0.0049 (7)	0.0019 (7)	−0.0042 (7)
C12	0.0287 (10)	0.0318 (11)	0.0307 (11)	0.0028 (8)	0.0056 (8)	−0.0027 (8)
C13	0.0281 (10)	0.0359 (11)	0.0314 (11)	−0.0020 (9)	0.0011 (8)	−0.0004 (9)
C14	0.0435 (14)	0.076 (2)	0.0645 (18)	−0.0022 (14)	−0.0170 (13)	0.0193 (15)
C15	0.0366 (13)	0.074 (2)	0.079 (2)	−0.0156 (13)	−0.0038 (13)	0.0177 (16)

### (III) N-[3-(Dimethylamino)propyl]-1-isopentyl-4-nitro-1H-pyrrole-2-carboxamide Geometric parameters (Å, º)

**Table:**

O1—N2	1.2325 (19)	C6—H6B	0.9900
O2—N2	1.2375 (19)	C7—C8	1.520 (3)
O3—C10	1.236 (2)	C7—C9	1.535 (3)
N1—C1	1.350 (2)	C7—H7	1.0000
N1—C4	1.388 (2)	C8—H8A	0.9800
N1—C5	1.471 (2)	C8—H8B	0.9800
N2—C2	1.424 (2)	C8—H8C	0.9800
N3—C10	1.338 (2)	C9—H9A	0.9800
N3—C11	1.456 (2)	C9—H9B	0.9800
N3—H1N	0.908 (9)	C9—H9C	0.9800
N4—C15	1.443 (3)	C11—C12	1.515 (3)
N4—C14	1.453 (3)	C11—H11A	0.9900
N4—C13	1.463 (3)	C11—H11B	0.9900
C1—C2	1.378 (2)	C12—C13	1.511 (3)
C1—H1	0.9500	C12—H12A	0.9900
C2—C3	1.404 (2)	C12—H12B	0.9900
C3—C4	1.369 (2)	C13—H13A	0.9900
C3—H3	0.9500	C13—H13B	0.9900
C4—C10	1.480 (2)	C14—H14A	0.9800
C5—C6	1.523 (2)	C14—H14B	0.9800
C5—H5A	0.9900	C14—H14C	0.9800
C5—H5B	0.9900	C15—H15A	0.9800
C6—C7	1.524 (2)	C15—H15B	0.9800
C6—H6A	0.9900	C15—H15C	0.9800
			
C1—N1—C4	109.28 (14)	H8A—C8—H8B	109.5
C1—N1—C5	123.63 (14)	C7—C8—H8C	109.5
C4—N1—C5	126.63 (14)	H8A—C8—H8C	109.5
O1—N2—O2	123.30 (15)	H8B—C8—H8C	109.5
O1—N2—C2	118.82 (15)	C7—C9—H9A	109.5
O2—N2—C2	117.88 (15)	C7—C9—H9B	109.5
C10—N3—C11	122.14 (15)	H9A—C9—H9B	109.5
C10—N3—H1N	117.1 (13)	C7—C9—H9C	109.5
C11—N3—H1N	120.6 (13)	H9A—C9—H9C	109.5
C15—N4—C14	109.9 (2)	H9B—C9—H9C	109.5
C15—N4—C13	112.51 (19)	O3—C10—N3	124.00 (16)
C14—N4—C13	110.86 (19)	O3—C10—C4	122.17 (16)
N1—C1—C2	107.04 (15)	N3—C10—C4	113.82 (15)
N1—C1—H1	126.5	N3—C11—C12	114.51 (15)
C2—C1—H1	126.5	N3—C11—H11A	108.6
C1—C2—C3	109.31 (15)	C12—C11—H11A	108.6
C1—C2—N2	124.59 (15)	N3—C11—H11B	108.6
C3—C2—N2	126.09 (15)	C12—C11—H11B	108.6
C4—C3—C2	105.77 (15)	H11A—C11—H11B	107.6
C4—C3—H3	127.1	C13—C12—C11	113.83 (16)
C2—C3—H3	127.1	C13—C12—H12A	108.8
C3—C4—N1	108.58 (15)	C11—C12—H12A	108.8
C3—C4—C10	128.54 (16)	C13—C12—H12B	108.8
N1—C4—C10	122.87 (15)	C11—C12—H12B	108.8
N1—C5—C6	110.60 (14)	H12A—C12—H12B	107.7
N1—C5—H5A	109.5	N4—C13—C12	112.04 (17)
C6—C5—H5A	109.5	N4—C13—H13A	109.2
N1—C5—H5B	109.5	C12—C13—H13A	109.2
C6—C5—H5B	109.5	N4—C13—H13B	109.2
H5A—C5—H5B	108.1	C12—C13—H13B	109.2
C5—C6—C7	113.79 (15)	H13A—C13—H13B	107.9
C5—C6—H6A	108.8	N4—C14—H14A	109.5
C7—C6—H6A	108.8	N4—C14—H14B	109.5
C5—C6—H6B	108.8	H14A—C14—H14B	109.5
C7—C6—H6B	108.8	N4—C14—H14C	109.5
H6A—C6—H6B	107.7	H14A—C14—H14C	109.5
C8—C7—C6	112.21 (17)	H14B—C14—H14C	109.5
C8—C7—C9	110.58 (19)	N4—C15—H15A	109.5
C6—C7—C9	109.48 (17)	N4—C15—H15B	109.5
C8—C7—H7	108.1	H15A—C15—H15B	109.5
C6—C7—H7	108.1	N4—C15—H15C	109.5
C9—C7—H7	108.1	H15A—C15—H15C	109.5
C7—C8—H8A	109.5	H15B—C15—H15C	109.5
C7—C8—H8B	109.5		
			
C4—N1—C1—C2	−0.96 (18)	C1—N1—C5—C6	98.60 (19)
C5—N1—C1—C2	−173.61 (14)	C4—N1—C5—C6	−72.7 (2)
N1—C1—C2—C3	0.21 (18)	N1—C5—C6—C7	179.02 (15)
N1—C1—C2—N2	−178.42 (15)	C5—C6—C7—C8	61.5 (2)
O1—N2—C2—C1	−0.8 (2)	C5—C6—C7—C9	−175.33 (18)
O2—N2—C2—C1	179.34 (15)	C11—N3—C10—O3	−7.4 (3)
O1—N2—C2—C3	−179.22 (15)	C11—N3—C10—C4	171.01 (14)
O2—N2—C2—C3	0.9 (3)	C3—C4—C10—O3	148.08 (18)
C1—C2—C3—C4	0.63 (18)	N1—C4—C10—O3	−30.3 (2)
N2—C2—C3—C4	179.23 (15)	C3—C4—C10—N3	−30.4 (2)
C2—C3—C4—N1	−1.20 (18)	N1—C4—C10—N3	151.23 (15)
C2—C3—C4—C10	−179.77 (15)	C10—N3—C11—C12	90.8 (2)
C1—N1—C4—C3	1.39 (18)	N3—C11—C12—C13	64.5 (2)
C5—N1—C4—C3	173.75 (15)	C15—N4—C13—C12	68.4 (3)
C1—N1—C4—C10	−179.95 (14)	C14—N4—C13—C12	−168.0 (2)
C5—N1—C4—C10	−7.6 (2)	C11—C12—C13—N4	159.69 (17)

### (III) N-[3-(Dimethylamino)propyl]-1-isopentyl-4-nitro-1H-pyrrole-2-carboxamide Hydrogen-bond geometry (Å, º)

**Table:**

*D*—H···*A*	*D*—H	H···*A*	*D*···*A*	*D*—H···*A*
N3—H1*N*···O3^i^	0.91 (1)	2.01 (1)	2.895 (2)	165 (2)
C5—H5*A*···O2^ii^	0.99	2.54	3.460 (2)	154

Symmetry codes: (i) *x*, −*y*+3/2, *z*+1/2; (ii) −*x*+1, *y*+1/2, −*z*+1/2.

### (IV) 1-(3-Azidopropyl)-4-(1-methyl-4-nitro-1H-pyrrole-2-carboxamido)-N-[2-(morpholin-4-yl)ethyl]-1H-pyrrole-2-carboxamide Crystal data

**Table:**

C_20_H_27_N_9_O_5_	*F*(000) = 1000
*M**_r_* = 473.51	*D*_x_ = 1.413 Mg m^−^^3^
Monoclinic, *P*2_1_/*c*	Mo *K*α radiation, λ = 0.71073 Å
Hall symbol: -P 2ybc	Cell parameters from 4332 reflections
*a* = 11.2809 (4) Å	θ = 3.3–29.5°
*b* = 16.4528 (6) Å	µ = 0.11 mm^−^^1^
*c* = 12.5130 (5) Å	*T* = 123 K
β = 106.542 (4)°	Plate, colourless
*V* = 2226.32 (14) Å^3^	0.30 × 0.28 × 0.03 mm
*Z* = 4	

### (IV) 1-(3-Azidopropyl)-4-(1-methyl-4-nitro-1H-pyrrole-2-carboxamido)-N-[2-(morpholin-4-yl)ethyl]-1H-pyrrole-2-carboxamide Data collection

**Table:**

Oxford Diffraction Xcalibur E diffractometer	4852 independent reflections
Radiation source: fine-focus sealed tube	3295 reflections with *I* > 2σ(*I*)
Graphite monochromator	*R*_int_ = 0.038
ω scans	θ_max_ = 27.0°, θ_min_ = 3.3°
Absorption correction: multi-scan (CrysAlis PRO; Oxford Diffraction, 2010)	*h* = −14→14
*T*_min_ = 0.828, *T*_max_ = 1.000	*k* = −21→20
14949 measured reflections	*l* = −15→15

### (IV) 1-(3-Azidopropyl)-4-(1-methyl-4-nitro-1H-pyrrole-2-carboxamido)-N-[2-(morpholin-4-yl)ethyl]-1H-pyrrole-2-carboxamide Refinement

**Table:**

Refinement on *F*^2^	Primary atom site location: structure-invariant direct methods
Least-squares matrix: full	Secondary atom site location: difference Fourier map
*R*[*F*^2^ > 2σ(*F*^2^)] = 0.049	Hydrogen site location: inferred from neighbouring sites
*wR*(*F*^2^) = 0.128	H atoms treated by a mixture of independent and constrained refinement
*S* = 1.03	*w* = 1/[σ^2^(*F*_o_^2^) + (0.0544*P*)^2^ + 0.8101*P*] where *P* = (*F*_o_^2^ + 2*F*_c_^2^)/3
4852 reflections	(Δ/σ)_max_ < 0.001
316 parameters	Δρ_max_ = 0.29 e Å^−^^3^
0 restraints	Δρ_min_ = −0.32 e Å^−^^3^

### (IV) 1-(3-Azidopropyl)-4-(1-methyl-4-nitro-1H-pyrrole-2-carboxamido)-N-[2-(morpholin-4-yl)ethyl]-1H-pyrrole-2-carboxamide Special details

**Table:**

Geometry. All s.u.'s (except the s.u. in the dihedral angle between two l.s. planes) are estimated using the full covariance matrix. The cell s.u.'s are taken into account individually in the estimation of s.u.'s in distances, angles and torsion angles; correlations between s.u.'s in cell parameters are only used when they are defined by crystal symmetry. An approximate (isotropic) treatment of cell s.u.'s is used for estimating s.u.'s involving l.s. planes.
Refinement. Refinement of F^2^ against ALL reflections. The weighted R-factor wR and goodness of fit S are based on F^2^, conventional R-factors R are based on F, with F set to zero for negative F^2^. The threshold expression of F^2^ > 2σ(F^2^) is used only for calculating R-factors(gt) etc. and is not relevant to the choice of reflections for refinement. R-factors based on F^2^ are statistically about twice as large as those based on F, and R- factors based on ALL data will be even larger.

### (IV) 1-(3-Azidopropyl)-4-(1-methyl-4-nitro-1H-pyrrole-2-carboxamido)-N-[2-(morpholin-4-yl)ethyl]-1H-pyrrole-2-carboxamide Fractional atomic coordinates and isotropic or equivalent isotropic displacement parameters (Å^2^)

**Table:**

	*x*	*y*	*z*	*U*_iso_*/*U*_eq_	
O1	1.41300 (15)	0.56045 (11)	1.22578 (13)	0.0398 (4)	
O2	0.98973 (13)	0.34257 (9)	0.69343 (11)	0.0258 (3)	
O3	0.74904 (13)	−0.00393 (9)	0.86469 (11)	0.0283 (4)	
O4	0.97289 (14)	−0.07180 (11)	1.39704 (12)	0.0368 (4)	
O5	0.86665 (15)	−0.18352 (10)	1.36804 (12)	0.0340 (4)	
N1	1.30194 (15)	0.45013 (11)	1.04738 (14)	0.0241 (4)	
N2	1.09195 (16)	0.35509 (11)	0.87526 (14)	0.0246 (4)	
N3	0.84913 (15)	0.20896 (10)	0.74083 (13)	0.0200 (4)	
N4	0.88041 (15)	0.08236 (11)	0.98173 (14)	0.0198 (4)	
N5	0.76427 (15)	−0.11643 (11)	1.04131 (13)	0.0212 (4)	
N6	0.90337 (16)	−0.12010 (12)	1.33493 (14)	0.0261 (4)	
N7	0.53925 (18)	0.24820 (15)	0.46339 (15)	0.0422 (6)	
N8	0.49433 (18)	0.24723 (13)	0.36178 (16)	0.0379 (5)	
N9	0.4479 (2)	0.24057 (16)	0.26908 (19)	0.0575 (7)	
C1	1.2953 (2)	0.57601 (15)	1.14934 (18)	0.0345 (6)	
H1A	1.2826	0.6354	1.1404	0.041*	
H1B	1.2299	0.5537	1.1795	0.041*	
C2	1.2846 (2)	0.53840 (13)	1.03754 (17)	0.0272 (5)	
H2A	1.2020	0.5506	0.9861	0.033*	
H2B	1.3476	0.5623	1.0058	0.033*	
C3	1.42227 (19)	0.43408 (15)	1.12873 (18)	0.0312 (5)	
H3A	1.4892	0.4540	1.0987	0.037*	
H3B	1.4331	0.3747	1.1406	0.037*	
C4	1.4324 (2)	0.47497 (16)	1.23829 (19)	0.0373 (6)	
H4A	1.3704	0.4513	1.2716	0.045*	
H4B	1.5154	0.4645	1.2899	0.045*	
C5	1.30008 (19)	0.41485 (14)	0.93966 (17)	0.0271 (5)	
H5A	1.3284	0.3577	0.9515	0.032*	
H5B	1.3598	0.4447	0.9097	0.032*	
C6	1.1751 (2)	0.41652 (14)	0.85378 (17)	0.0280 (5)	
H6A	1.1376	0.4709	0.8539	0.034*	
H6B	1.1860	0.4073	0.7789	0.034*	
C7	1.00628 (18)	0.32014 (12)	0.79114 (15)	0.0194 (4)	
C8	0.93886 (17)	0.25098 (12)	0.81991 (15)	0.0180 (4)	
C9	0.96041 (17)	0.20919 (12)	0.91950 (15)	0.0187 (4)	
H9	1.0175	0.2240	0.9885	0.022*	
C10	0.88249 (17)	0.14092 (12)	0.90005 (15)	0.0178 (4)	
C11	0.81442 (18)	0.14275 (13)	0.78955 (15)	0.0212 (4)	
H11	0.7534	0.1042	0.7535	0.025*	
C12	0.81419 (17)	0.01299 (13)	0.95888 (15)	0.0191 (4)	
C13	0.82387 (17)	−0.04175 (12)	1.05467 (16)	0.0196 (4)	
C14	0.88497 (18)	−0.03205 (13)	1.16576 (16)	0.0211 (4)	
H14	0.9339	0.0131	1.1995	0.025*	
C15	0.86064 (18)	−0.10201 (13)	1.21902 (15)	0.0209 (4)	
C16	0.78689 (18)	−0.15307 (13)	1.14111 (16)	0.0226 (4)	
H16	0.7573	−0.2049	1.1552	0.027*	
C17	0.6959 (2)	−0.15598 (14)	0.93648 (17)	0.0296 (5)	
H17A	0.6654	−0.2090	0.9529	0.044*	
H17B	0.7509	−0.1636	0.8892	0.044*	
H17C	0.6258	−0.1218	0.8975	0.044*	
C18	0.78428 (19)	0.23250 (13)	0.62593 (15)	0.0233 (5)	
H18A	0.7383	0.1850	0.5861	0.028*	
H18B	0.8458	0.2488	0.5872	0.028*	
C19	0.69467 (19)	0.30212 (14)	0.62105 (16)	0.0266 (5)	
H19A	0.6400	0.2886	0.6679	0.032*	
H19B	0.7419	0.3515	0.6524	0.032*	
C20	0.6165 (2)	0.31976 (15)	0.50420 (18)	0.0321 (5)	
H20A	0.5637	0.3679	0.5039	0.039*	
H20B	0.6699	0.3312	0.4555	0.039*	
H1N	1.094 (2)	0.3458 (14)	0.9407 (19)	0.025 (6)*	
H2N	0.922 (2)	0.0947 (15)	1.051 (2)	0.035 (7)*	

### (IV) 1-(3-Azidopropyl)-4-(1-methyl-4-nitro-1H-pyrrole-2-carboxamido)-N-[2-(morpholin-4-yl)ethyl]-1H-pyrrole-2-carboxamide Atomic displacement parameters (Å^2^)

**Table:**

	*U*^11^	*U*^22^	*U*^33^	*U*^12^	*U*^13^	*U*^23^
O1	0.0397 (9)	0.0420 (11)	0.0339 (9)	−0.0102 (9)	0.0042 (7)	−0.0090 (8)
O2	0.0354 (8)	0.0221 (8)	0.0175 (7)	−0.0041 (7)	0.0036 (6)	0.0044 (6)
O3	0.0364 (8)	0.0263 (8)	0.0184 (8)	−0.0069 (7)	0.0017 (6)	0.0004 (6)
O4	0.0357 (9)	0.0497 (11)	0.0211 (8)	−0.0107 (9)	0.0021 (7)	0.0016 (8)
O5	0.0510 (10)	0.0254 (9)	0.0273 (8)	0.0052 (8)	0.0139 (7)	0.0106 (7)
N1	0.0229 (8)	0.0221 (10)	0.0252 (9)	−0.0034 (8)	0.0037 (7)	0.0000 (8)
N2	0.0331 (10)	0.0261 (10)	0.0145 (9)	−0.0090 (9)	0.0067 (7)	−0.0014 (8)
N3	0.0263 (9)	0.0180 (9)	0.0140 (8)	−0.0012 (8)	0.0030 (7)	0.0003 (7)
N4	0.0260 (9)	0.0178 (9)	0.0146 (9)	−0.0011 (8)	0.0045 (7)	0.0013 (7)
N5	0.0253 (8)	0.0180 (9)	0.0203 (9)	−0.0001 (8)	0.0067 (7)	0.0005 (7)
N6	0.0280 (9)	0.0294 (11)	0.0215 (9)	0.0081 (9)	0.0081 (8)	0.0067 (8)
N7	0.0405 (11)	0.0530 (15)	0.0259 (11)	−0.0132 (11)	−0.0019 (9)	0.0015 (10)
N8	0.0345 (10)	0.0428 (13)	0.0322 (12)	0.0017 (10)	0.0028 (9)	−0.0026 (10)
N9	0.0693 (16)	0.0580 (17)	0.0339 (13)	0.0029 (14)	−0.0036 (12)	−0.0083 (11)
C1	0.0374 (12)	0.0333 (14)	0.0329 (13)	−0.0019 (12)	0.0098 (10)	−0.0066 (10)
C2	0.0289 (11)	0.0217 (12)	0.0307 (12)	−0.0011 (10)	0.0078 (9)	−0.0010 (9)
C3	0.0237 (10)	0.0291 (13)	0.0367 (13)	−0.0033 (10)	0.0018 (9)	0.0059 (10)
C4	0.0312 (12)	0.0444 (16)	0.0306 (13)	−0.0089 (12)	−0.0003 (10)	0.0073 (11)
C5	0.0280 (11)	0.0218 (11)	0.0320 (12)	−0.0047 (10)	0.0095 (9)	−0.0034 (9)
C6	0.0358 (12)	0.0243 (12)	0.0238 (11)	−0.0107 (10)	0.0082 (9)	−0.0011 (9)
C7	0.0233 (9)	0.0172 (10)	0.0182 (10)	0.0024 (9)	0.0069 (8)	−0.0012 (8)
C8	0.0220 (9)	0.0154 (10)	0.0162 (9)	0.0020 (9)	0.0047 (7)	−0.0035 (8)
C9	0.0209 (9)	0.0190 (10)	0.0161 (9)	0.0016 (9)	0.0053 (8)	−0.0022 (8)
C10	0.0220 (9)	0.0160 (10)	0.0157 (9)	0.0022 (8)	0.0057 (8)	0.0013 (8)
C11	0.0256 (10)	0.0178 (11)	0.0192 (10)	−0.0026 (9)	0.0048 (8)	0.0000 (8)
C12	0.0213 (9)	0.0188 (10)	0.0181 (10)	0.0023 (9)	0.0071 (8)	−0.0005 (8)
C13	0.0209 (9)	0.0190 (11)	0.0199 (10)	0.0026 (9)	0.0074 (8)	0.0006 (8)
C14	0.0235 (10)	0.0213 (11)	0.0188 (10)	0.0016 (9)	0.0064 (8)	0.0000 (8)
C15	0.0228 (10)	0.0224 (11)	0.0185 (10)	0.0041 (9)	0.0072 (8)	0.0027 (8)
C16	0.0280 (10)	0.0179 (11)	0.0245 (11)	0.0032 (9)	0.0118 (9)	0.0054 (8)
C17	0.0386 (12)	0.0230 (12)	0.0248 (11)	−0.0068 (11)	0.0052 (9)	−0.0013 (9)
C18	0.0300 (11)	0.0234 (11)	0.0133 (10)	−0.0020 (10)	0.0009 (8)	−0.0001 (8)
C19	0.0264 (10)	0.0290 (13)	0.0230 (11)	0.0014 (10)	0.0045 (9)	0.0013 (9)
C20	0.0266 (11)	0.0348 (14)	0.0312 (12)	−0.0027 (11)	0.0023 (9)	0.0072 (10)

### (IV) 1-(3-Azidopropyl)-4-(1-methyl-4-nitro-1H-pyrrole-2-carboxamido)-N-[2-(morpholin-4-yl)ethyl]-1H-pyrrole-2-carboxamide Geometric parameters (Å, º)

**Table:**

O1—C1	1.421 (3)	C3—H3B	0.9900
O1—C4	1.425 (3)	C4—H4A	0.9900
O2—C7	1.239 (2)	C4—H4B	0.9900
O3—C12	1.230 (2)	C5—C6	1.510 (3)
O4—N6	1.226 (2)	C5—H5A	0.9900
O5—N6	1.237 (2)	C5—H5B	0.9900
N1—C5	1.462 (3)	C6—H6A	0.9900
N1—C2	1.466 (3)	C6—H6B	0.9900
N1—C3	1.470 (3)	C7—C8	1.469 (3)
N2—C7	1.339 (3)	C8—C9	1.383 (3)
N2—C6	1.455 (3)	C9—C10	1.404 (3)
N2—H1N	0.83 (2)	C9—H9	0.9500
N3—C11	1.359 (3)	C10—C11	1.378 (3)
N3—C8	1.383 (2)	C11—H11	0.9500
N3—C18	1.467 (2)	C12—C13	1.478 (3)
N4—C12	1.349 (3)	C13—C14	1.374 (3)
N4—C10	1.410 (2)	C14—C15	1.396 (3)
N4—H2N	0.88 (2)	C14—H14	0.9500
N5—C16	1.344 (2)	C15—C16	1.374 (3)
N5—C13	1.388 (3)	C16—H16	0.9500
N5—C17	1.471 (3)	C17—H17A	0.9800
N6—C15	1.424 (2)	C17—H17B	0.9800
N7—N8	1.227 (3)	C17—H17C	0.9800
N7—C20	1.467 (3)	C18—C19	1.517 (3)
N8—N9	1.134 (3)	C18—H18A	0.9900
C1—C2	1.503 (3)	C18—H18B	0.9900
C1—H1A	0.9900	C19—C20	1.505 (3)
C1—H1B	0.9900	C19—H19A	0.9900
C2—H2A	0.9900	C19—H19B	0.9900
C2—H2B	0.9900	C20—H20A	0.9900
C3—C4	1.502 (3)	C20—H20B	0.9900
C3—H3A	0.9900		
			
C1—O1—C4	109.65 (18)	H6A—C6—H6B	107.9
C5—N1—C2	110.46 (17)	O2—C7—N2	121.32 (19)
C5—N1—C3	109.57 (17)	O2—C7—C8	121.96 (17)
C2—N1—C3	108.13 (17)	N2—C7—C8	116.62 (16)
C7—N2—C6	120.80 (17)	N3—C8—C9	107.54 (17)
C7—N2—H1N	120.7 (16)	N3—C8—C7	122.45 (16)
C6—N2—H1N	118.2 (16)	C9—C8—C7	129.45 (17)
C11—N3—C8	109.00 (16)	C8—C9—C10	107.51 (17)
C11—N3—C18	121.61 (16)	C8—C9—H9	126.2
C8—N3—C18	128.93 (17)	C10—C9—H9	126.2
C12—N4—C10	123.17 (17)	C11—C10—C9	107.40 (17)
C12—N4—H2N	120.6 (16)	C11—C10—N4	128.53 (18)
C10—N4—H2N	116.1 (16)	C9—C10—N4	124.06 (17)
C16—N5—C13	109.14 (17)	N3—C11—C10	108.55 (17)
C16—N5—C17	122.87 (18)	N3—C11—H11	125.7
C13—N5—C17	127.76 (17)	C10—C11—H11	125.7
O4—N6—O5	123.31 (17)	O3—C12—N4	122.55 (18)
O4—N6—C15	118.70 (18)	O3—C12—C13	121.67 (19)
O5—N6—C15	117.98 (18)	N4—C12—C13	115.78 (17)
N8—N7—C20	113.6 (2)	C14—C13—N5	108.13 (17)
N9—N8—N7	174.3 (3)	C14—C13—C12	130.51 (19)
O1—C1—C2	111.39 (19)	N5—C13—C12	121.36 (17)
O1—C1—H1A	109.3	C13—C14—C15	106.08 (18)
C2—C1—H1A	109.3	C13—C14—H14	127.0
O1—C1—H1B	109.3	C15—C14—H14	127.0
C2—C1—H1B	109.3	C16—C15—C14	109.09 (17)
H1A—C1—H1B	108.0	C16—C15—N6	123.80 (19)
N1—C2—C1	110.88 (18)	C14—C15—N6	127.11 (19)
N1—C2—H2A	109.5	N5—C16—C15	107.55 (19)
C1—C2—H2A	109.5	N5—C16—H16	126.2
N1—C2—H2B	109.5	C15—C16—H16	126.2
C1—C2—H2B	109.5	N5—C17—H17A	109.5
H2A—C2—H2B	108.1	N5—C17—H17B	109.5
N1—C3—C4	111.51 (19)	H17A—C17—H17B	109.5
N1—C3—H3A	109.3	N5—C17—H17C	109.5
C4—C3—H3A	109.3	H17A—C17—H17C	109.5
N1—C3—H3B	109.3	H17B—C17—H17C	109.5
C4—C3—H3B	109.3	N3—C18—C19	112.29 (16)
H3A—C3—H3B	108.0	N3—C18—H18A	109.1
O1—C4—C3	111.86 (19)	C19—C18—H18A	109.1
O1—C4—H4A	109.2	N3—C18—H18B	109.1
C3—C4—H4A	109.2	C19—C18—H18B	109.1
O1—C4—H4B	109.2	H18A—C18—H18B	107.9
C3—C4—H4B	109.2	C20—C19—C18	112.59 (18)
H4A—C4—H4B	107.9	C20—C19—H19A	109.1
N1—C5—C6	114.48 (18)	C18—C19—H19A	109.1
N1—C5—H5A	108.6	C20—C19—H19B	109.1
C6—C5—H5A	108.6	C18—C19—H19B	109.1
N1—C5—H5B	108.6	H19A—C19—H19B	107.8
C6—C5—H5B	108.6	N7—C20—C19	108.00 (19)
H5A—C5—H5B	107.6	N7—C20—H20A	110.1
N2—C6—C5	112.10 (18)	C19—C20—H20A	110.1
N2—C6—H6A	109.2	N7—C20—H20B	110.1
C5—C6—H6A	109.2	C19—C20—H20B	110.1
N2—C6—H6B	109.2	H20A—C20—H20B	108.4
C5—C6—H6B	109.2		
			
C20—N7—N8—N9	−177 (3)	C18—N3—C11—C10	−173.55 (17)
C4—O1—C1—C2	−58.5 (2)	C9—C10—C11—N3	0.7 (2)
C5—N1—C2—C1	−175.98 (17)	N4—C10—C11—N3	−178.01 (18)
C3—N1—C2—C1	−56.1 (2)	C10—N4—C12—O3	−0.9 (3)
O1—C1—C2—N1	59.5 (2)	C10—N4—C12—C13	179.47 (17)
C5—N1—C3—C4	175.41 (19)	C16—N5—C13—C14	0.0 (2)
C2—N1—C3—C4	55.0 (2)	C17—N5—C13—C14	−174.61 (18)
C1—O1—C4—C3	57.3 (2)	C16—N5—C13—C12	−179.14 (17)
N1—C3—C4—O1	−56.8 (3)	C17—N5—C13—C12	6.2 (3)
C2—N1—C5—C6	−69.5 (2)	O3—C12—C13—C14	−175.9 (2)
C3—N1—C5—C6	171.44 (18)	N4—C12—C13—C14	3.7 (3)
C7—N2—C6—C5	−148.35 (19)	O3—C12—C13—N5	3.1 (3)
N1—C5—C6—N2	−75.6 (2)	N4—C12—C13—N5	−177.36 (17)
C6—N2—C7—O2	−4.8 (3)	N5—C13—C14—C15	−0.3 (2)
C6—N2—C7—C8	171.58 (18)	C12—C13—C14—C15	178.8 (2)
C11—N3—C8—C9	0.4 (2)	C13—C14—C15—C16	0.4 (2)
C18—N3—C8—C9	172.56 (18)	C13—C14—C15—N6	−179.62 (18)
C11—N3—C8—C7	172.58 (17)	O4—N6—C15—C16	177.15 (19)
C18—N3—C8—C7	−15.3 (3)	O5—N6—C15—C16	−3.9 (3)
O2—C7—C8—N3	−2.9 (3)	O4—N6—C15—C14	−2.8 (3)
N2—C7—C8—N3	−179.27 (18)	O5—N6—C15—C14	176.11 (19)
O2—C7—C8—C9	167.3 (2)	C13—N5—C16—C15	0.2 (2)
N2—C7—C8—C9	−9.0 (3)	C17—N5—C16—C15	175.17 (17)
N3—C8—C9—C10	0.0 (2)	C14—C15—C16—N5	−0.4 (2)
C7—C8—C9—C10	−171.38 (19)	N6—C15—C16—N5	179.63 (17)
C8—C9—C10—C11	−0.5 (2)	C11—N3—C18—C19	101.0 (2)
C8—C9—C10—N4	178.35 (17)	C8—N3—C18—C19	−70.2 (3)
C12—N4—C10—C11	5.5 (3)	N3—C18—C19—C20	−172.88 (17)
C12—N4—C10—C9	−173.06 (18)	N8—N7—C20—C19	−163.3 (2)
C8—N3—C11—C10	−0.7 (2)	C18—C19—C20—N7	63.8 (2)

### (IV) 1-(3-Azidopropyl)-4-(1-methyl-4-nitro-1H-pyrrole-2-carboxamido)-N-[2-(morpholin-4-yl)ethyl]-1H-pyrrole-2-carboxamide Hydrogen-bond geometry (Å, º)

**Table:**

*D*—H···*A*	*D*—H	H···*A*	*D*···*A*	*D*—H···*A*
N2—H1*N*···O5^i^	0.83 (2)	2.36 (2)	3.176 (2)	171 (2)
N4—H2*N*···O2^ii^	0.88 (2)	2.02 (2)	2.864 (2)	162 (2)
C2—H2*A*···O4^iii^	0.99	2.53	3.498 (3)	165
C6—H6*B*···O3^iv^	0.99	2.58	3.354 (3)	135
C9—H9···O5^i^	0.95	2.43	3.322 (2)	156
C14—H14···O2^ii^	0.95	2.46	3.317 (3)	149

Symmetry codes: (i) −*x*+2, *y*+1/2, −*z*+5/2; (ii) *x*, −*y*+1/2, *z*+1/2; (iii) *x*, −*y*+1/2, *z*−1/2; (iv) −*x*+2, *y*+1/2, −*z*+3/2.
